# Biomass-Based Antifouling Coatings: Mechanisms, Materials, Applications, and Emerging Sustainable Strategies

**DOI:** 10.1007/s40820-026-02137-4

**Published:** 2026-03-27

**Authors:** Yudi Wei, Dominik Maršík, Petter Paulsen Thoresen, Leonidas Matsakas, Yijun Shi

**Affiliations:** 1 Division of Machine Elements, Luleå University of Technology, 97187 Luleå, Sweden; 2 Biochemical Process Engineering, Division of Chemical Engineering, Department of Civil, Environmental and Natural Resources Engineering, Luleå University of Technology, 971-87 Luleå, Sweden

**Keywords:** Biomass-based antifouling coatings, Antifouling mechanisms, Crosslinking strategies, Marine and biomedical applications

## Abstract

Biofouling remains a critical challenge across marine, medical, and industrial sectors, driving the need for sustainable and eco-friendly antifouling coatings. Biomass-based materials, including lignin, tannins, betaines, polysaccharides, and proteins, have emerged as promising candidates due to their biocompatibility, biodegradability, and diverse antifouling mechanisms. These materials inhibit biofouling through mechanisms such as hydration layer formation, surface charge repulsion, hydrophobic foul-release, and biocidal effects, offering effective resistance to protein adsorption, bacterial attachment, and biofilm formation. This review provides a comprehensive overview of recent advancements in biomass-based antifouling coatings, focusing on their antifouling mechanisms, synthesis methods, and applications. Key challenges concerning mechanical durability and scalability are explored, along with potential approaches to enhance the development of sustainable antifouling technologies.

## Highlights

- This review systematically categorizes biomass-based antifouling coatings based on lignin, tannins, betaines, polysaccharides and proteins, highlighting how their intrinsic chemistries contribute to antifouling interfaces.
- The review clarifies how hydration layers, foul-release interfaces, surface charge regulation and biocidal actions collectively contribute to antifouling performance.
- Key chemical and physical crosslinking strategies are compared to highlight their roles in enhancing mechanical integrity and long-term stability.
- Critical bottlenecks in durability, adhesion, validation and scalability are identified, and potential routes for future technological development are outlined.

## Graphical abstract

**Figure:**
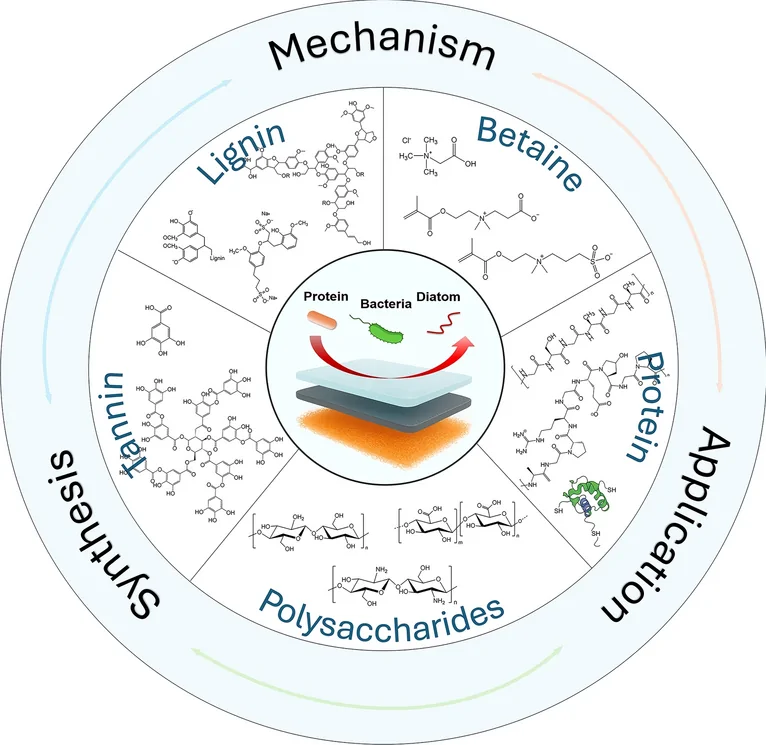

## Introduction

Biofouling refers to the undesirable accumulation and attachment of biological material on a man-made surface [1]. In the marine field, biofouling accelerates hull corrosion [2, 3], increases resistance and fuel consumption [4–6], and imposes a huge economic burden on the global shipping industry [7, 8]. The United Nations Conference on Trade and Development (UNCTAD) reports that the shipping industry needs to invest an additional $80 billion to $280 billion each year to achieve its 2050 carbon reduction goals. Similar concerns exist in the medical field, where biofilms formed on medical devices can cause serious healthcare-associated infections (HAIs). According to a 2022 report by the World Health Organization (WHO), approximately 7 out of every 100 hospitalized patients in high-income countries contract nosocomial infections during their hospital stay; in low- and middle-income countries, this proportion rises to 15 out of every 100 patients [9].

**Fig. 1:**
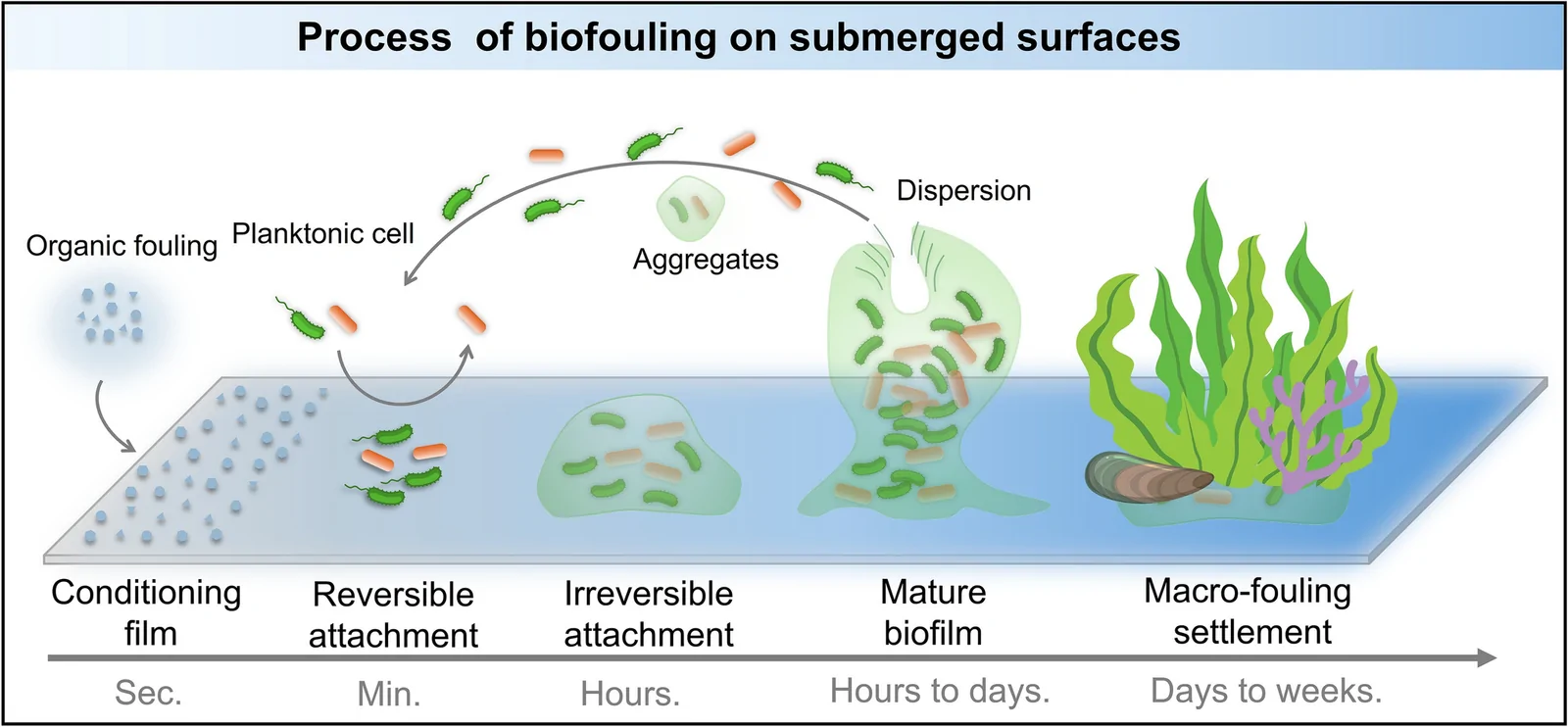
Schematic illustration of biofouling development

The formation of biofouling depends on environmental conditions, yet surfaces exposed to aquatic environments are readily colonized by microorganisms [10]. Time-dependent studies conducted in marine environments have demonstrated that biomacromolecules adhere to surfaces almost immediately upon contact with seawater (Fig. 1) [11, 12]. These are proteins and glycoproteins that form a conditioning film [13, 14], initiating the biofouling process, which is typically described as a cascade of successive stages. The adsorption of these organic molecules is followed by microbial attachment and colonization, leading to the formation of a biofilm which provides a suitable basis for the subsequent attachment of larger, yet still microscopic propagules, including algal cells and zoospores. Subsequently, macroscopic organisms, such as invertebrates, may settle and grow, marking the final stage of the biofouling succession [15].

From the perspective of this review, the most critical step in controlling biofouling appears to be the suppression of the initial stages of the cascade process, specifically the formation of the conditioning film and the subsequent microbial adhesion leading to biofilm development. Polymer-based coatings are particularly effective at short distances, especially within the range of direct contact between the biological agent and the surface, thereby preventing biofilm establishment and, consequently, the development of macrofouling, which is the main contributor to substantial economic costs over the vessel’s service life. The conditioning film itself can alter surface properties and promote microbial adhesion [16]. When focusing on microorganisms and the development of biofilms, the process is often described in a general way as a cascade model consisting of five phases: the planktonic phase of bacteria, initial attachment, microcolony formation, biofilm maturation, and dispersion. The initial attachment phase is commonly divided into reversible and irreversible stages, as early interactions are typically weak and stable adhesion requires active cellular mechanisms involvement [17]. However, this model is not universally applicable and does not fully reflect the widespread nature of biofilms as a dominant form of microbial lifestyle [18]. To address these limitations, an extended model of biofilm formation has been proposed, which considers bacterial aggregates as biofilms regardless of whether they are attached to a surface. Adopting this perspective implies that ship hulls are exposed not only to individual planktonic cells, but also to biofilms themselves [19], an aspect that may strongly influence the rational design of antifouling coatings. Biological aggregates, whether surface-attached or free-floating, generally exhibit increased resistance due to the presence of extracellular polymeric substances (EPS). Cells embedded within EPS demonstrate higher tolerance to biocides and mechanical stress [20]. The mechanical properties of biofilms depend on several factors, including EPS composition, microbial community structure, nutrient availability, and environmental conditions, with EPS composition recognized as the primary determinant [21]. In addition, EPS facilitates intercellular chemical communication through quorum sensing, further supporting structural and functional integrity of the biofilm [22].

The EPS is mainly composed of exopolysaccharides, proteins, extracellular DNA (eDNA), lipids, and other biomolecules [23]. Structurally, exopolysaccharides can be classified as homoglycans, consisting of a single type of monosaccharide, or heteroglycans, composed of repeating units of different monosaccharides. In addition, polysaccharides may vary in molecular weight, the type of bonding between units, their sequence, branching pattern, and the presence of functional groups such as sulfates, phosphates, or carboxylates, which contribute to the overall negative charge of the polymer [24]. The structural variations of polysaccharides may be shaped by the environmental conditions in which the bacteria occur [25]. Structural variations are also observed in proteins. Proteins of halophilic bacteria are known for their high stability and are enriched in acidic amino acids on their surface, which support protein hydration [26]. Depending on external conditions, bacteria can modulate protein secretion [27], and membrane proteins may undergo structural and functional changes in response to salt concentration [28]. eDNA is considered another key component of EPS, contributing to its overall negative charge. It plays an important role in maintaining the structural integrity of multispecies biofilms, with its abundance reflecting not the prevalence of individual species, but the collective stabilizing function of the microbial community [29, 30].

Currently, several strategies are tested to inhibit biofilm formation [31, 32], including the prevention of bacterial adhesion [33], interference with quorum sensing [34], and disruption of the established biofilm matrix [35]. Inhibition of bacterial attachment through antifouling coatings remains a practical and cost-effective approach [36, 37]. A variety of synthetic antifouling coatings are currently available, including metal-based coatings, polymer-based coatings [38], fluoropolymer coatings [39, 40], hydrogel coatings [41, 42], and composite coatings [43–46]. Although these coatings exhibit effective antifouling performance, many of them pose environmental risk due to poor biodegradability and potential toxicity. Traditional coatings containing metal ions such as copper or organic biocides have therefore been strictly regulated, emphasizing the need for sustainable and environmentally benign alternatives.

Biomass-based antifouling coatings have attracted significant attention as a sustainable and environmentally friendly approach to mitigating biofouling [47, 48]. Derived from renewable biological sources, these coatings exhibit key advantages such as inherent biodegradability, biocompatibility, and reduced ecological impact. Their development aligns with the growing demand for eco-conscious alternatives to traditional antifouling strategies. Currently, biomass-based materials utilized in antifouling coatings can be broadly classified into the following categories:

(i) Lignin-based coatings exploit the natural antimicrobial and antioxidant properties of lignin while promoting the sustainable use of wood industry by-products from the wood pulping and paper industry.
(ii) Betaine-based coatings, including sulfobetaine and carboxybetaine, rely on their zwitterionic nature; while often synthesized, these structures are bio-inspired by osmoprotectants found in various plants, algae, and marine organisms.
(iii) Polysaccharide-based coatings, derived from abundant natural polymers such as chitosan (extracted from crustacean shells), alginate (derived from brown seaweed), and cellulose (the primary structural component of green plants).
(iv) Tannin-based coatings utilize polyphenolic compounds widely distributed in plant tissues such as bark, fruits, and leaves. These coatings disrupt microbial colonization and biofilm formation through their inherent bioactivity, offering a natural alternative to synthetic biocides.
(v) Protein-based coatings, utilizing structural proteins such as silk fibroin from silkworm cocoons and gelatin derived from animal collagen, contribute to antifouling through structural adaptability and surface modifications.

The fundamental antifouling principle underlying these biomass-based materials is their ability to hinder the initial interaction with biomacromolecules forming conditioning layer on surfaces during the biofouling process or to promote their easy removal through a range of intrinsic properties, including, but not limited to, low surface energy [49], formation of a surface hydration layer [50] and surface charge neutrality [51]. Moreover, surfaces of these biomaterials typically possess a high density of functional groups, such as amino (–NH_2_), carboxyl –COOH) and hydroxyl (–OH) groups, which enable versatile chemical modification and facilitate their application in various contexts, including antifouling self-healing coatings [52, 53], flexible smart sensors [54], and biocompatible medical devices [55].

This review provides a comprehensive overview of biomass-based antifouling coatings, emphasizing their antifouling mechanisms, synthesis strategies, and practical applications. While several reviews have discussed biomass-derived materials for antifouling applications, they often focus on specific biopolymers or limited fouling environments. This review adopts a mechanism-driven and application-specific perspective. By establishing a direct link between fundamental interfacial principles, quantitative performance benchmarks, and specific environmental requirements, we aim to provide a strategic design framework. This approach not only summarizes recent advances, but also addresses the practical trade-offs between mechanical durability, scalability, and long-term ecological safety, thereby offering a comprehensive roadmap for the transition from laboratory discovery to industrial deployment.

## Mechanisms of Antifouling in Biomass-Based Coatings

The antifouling performance of biomass-based coatings depends mainly on the ability to prevent the initial adhesion of organic molecules, proteins, and microorganisms to the surfaces. Generally, these coatings achieve antifouling through two main pathways that can be categorized into passive and active mechanisms: (i) passive anti-protein adhesion mechanisms, which generate non-fouling interfaces that repel proteins and cells, and (ii) active biocidal mechanisms, which may inhibit or destroy fouling organisms through active chemical or physical interference. The synergistic potential of combining these passive and active concepts represents a broad strategy toward sustainable and eco-friendly antifouling systems.

### Anti-Protein Adhesion Mechanism

The anti-protein adhesion mechanism operates primarily by minimizing the interfacial energy and preventing the adsorption of conditioning films, which are critical precursors for microbial colonization. So far, various physicochemical strategies have been developed to achieve this goal, as summarized below.

#### Hydrophilic Layer Formation

**Fig. 2:**
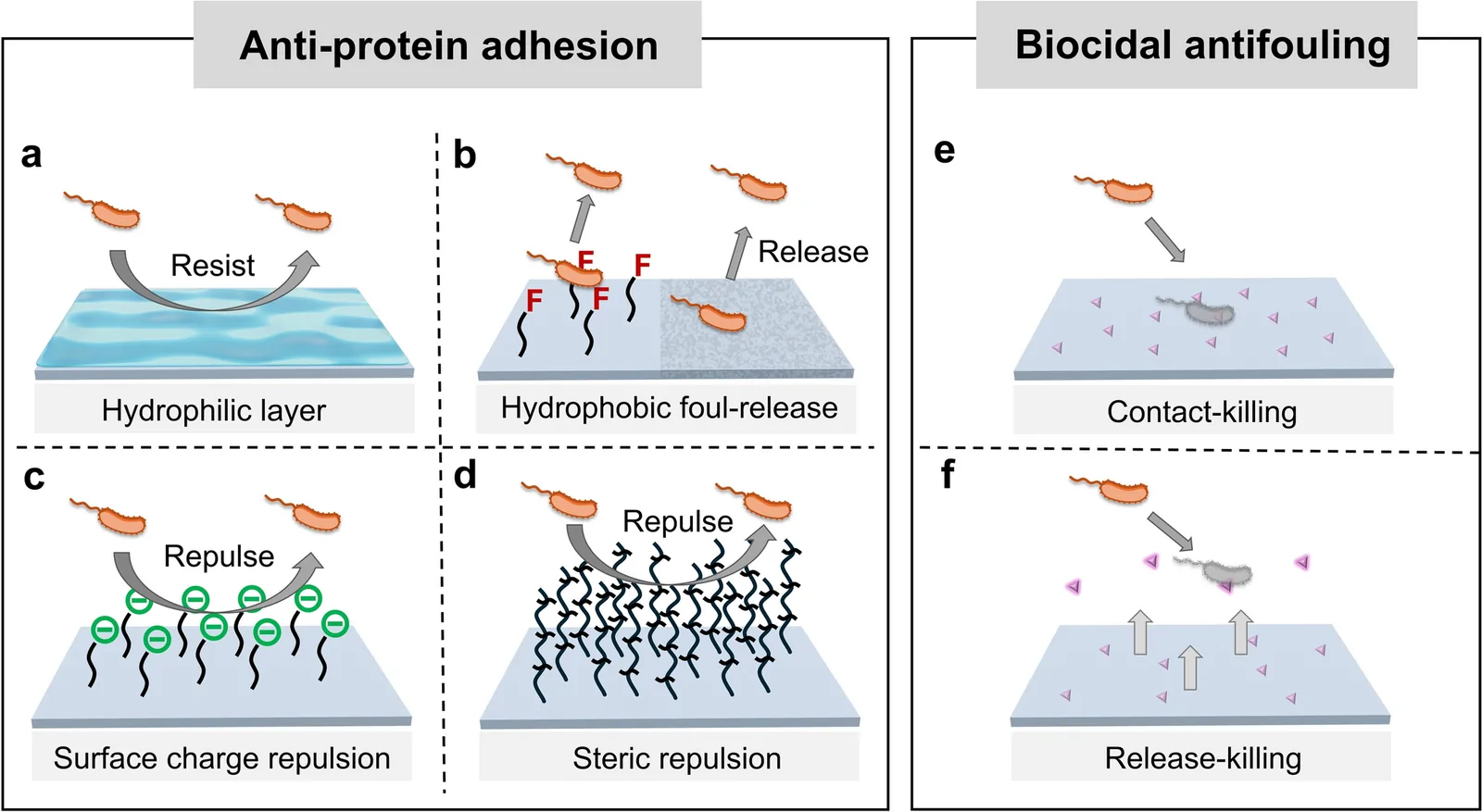
Schematic illustration of antifouling mechanisms. **a** Hydrophilic layer formation. **b** Hydrophobic foul-release (The ‘F’ represents fluorinated terminal groups). **c** Surface charge repulsion. **d** Steric repulsion. **e** Contact-killing antifouling. **f** Release-killing antifouling

Hydrophilic antifouling coatings work by forming a hydration layer at the surface (Fig. 2a). The layer provides both energetic and physical obstacles to protein adsorption and microbial attachment [56, 57]. Biomass-derived materials, such as polysaccharides, zwitterionic betaines, and polyphenols, are rich in hydrophilic functional groups, including –OH, –COOH, –NH_2_, and –SO_3_^−^, which can bind water molecules through hydrogen bonding. This bound water layer is structurally stable with extremely low interfacial free energy, effectively repelling biological macromolecules [58, 59]. For example, zwitterionic coatings utilize electrostatically balanced cationic and anionic groups to form a tightly bound hydration shell, allowing it to maintain charge neutrality while creating strong osmotic repulsion [60–62]. Similarly, polysaccharide-based coatings such as alginate and cellulose can retain substantial amounts of bound water, forming a dynamic yet stable barrier that minimizes protein adsorption and delays biofilm initiation [63, 64].

#### Hydrophobic Foul-release Mechanism

Unlike hydrophilic coatings, hydrophobic foul-release coatings take a different approach. They rely on low surface energy and weak interfacial adhesion, which allows fouling species to be removed easily. Surfaces with low-energy functional groups, such as –CF_3_, –CH_3_, or siloxane (-Si–O-Si-) structures, minimize the adhesion between foulants and the substrate (Fig. 2b, left part).

Biomass-based hydrophobic systems can be constructed in several ways, including chemical modification of natural polymers (for example, acylation or alkylation of polysaccharides, grafting of fatty acids or plant oils) or incorporation with silicone matrices [65–67]. In addition to chemical modification, surface topography greatly influences foul-release performance (Fig. 2b right part) [68]. Micro- and nanoscale or bioinspired surfaces [49, 69–71], mimicking lotus leaves, shark skin, or fish scales, enhance water repellence by trapping air pockets and minimizing the effective contact area. Similar topographies can be fabricated from biomass-based materials through templating or patterning [48], enabling sustainable hydrophobic and foul-release coating designs.

#### Surface Charge Repulsion

Surface charge modulation offers another effective route to antifouling (Fig. 2c). Since most proteins and bacterial cell walls carry negative charges under physiological conditions, negatively charged coatings can electrostatically repel them [72–74]. Biomass-based materials that contain carboxyl or sulfate groups, such as alginate, carboxylated cellulose, and lignosulfonate, present these negative charges. Yang et al. developed TEMPO-oxidized cellulose nanofiber layers by converting surface hydroxyl groups into carboxyl groups [75], thereby introducing tunable negative charges. The modified membranes exhibited strong electrostatic repulsion against negatively charged proteins, resulting in reduced adsorption.

#### Steric Repulsion

Steric repulsion rises when polymer chains extending from the surface (Fig. 2d), such as polymer brushes or long side-chain networks [76–79], generate an entropic barrier that hinders macromolecules from approaching the substrate. Hydrophilic polymer brushes, often derived from biomass components such as zwitterionic betaines [80, 81] and polysaccharide chains [82], confer high configurational entropy at the interface. When proteins or cells approach the surface, compression of these flexible chains creates a repulsive force that counteracts adsorption. The magnitude of steric repulsion depends on chain length, grafting density, and the degree of solvation; parameters that can be precisely tuned in biomass-derived systems through molecular design [83, 84].

### Biocidal Antifouling Mechanism

While anti-protein adhesion strategies focus on preventing fouling attachment, biocidal antifouling mechanisms take a more active approach by eliminating microorganisms that contact the coating surface. In biomass-based systems, these mechanisms generally fall into two categories: contact-killing and release-killing processes.

#### Contact-killing Antifouling

Contact-killing coatings destroy microorganisms upon direct physical or chemical interaction with the surface (Fig. 2e). Positively charged functional groups such as quaternary ammonium (–NR_4_^+^) or protonated amines (–NH_3_^+^) can interact with negatively charged bacterial envelopes. This electrostatic interaction causes structural disruption, leakage of intracellular contents, and cell death [85, 86]. Coatings based on chitosan are examples of this mechanism, as their cationic nature facilitates this effective electrostatic binding to bacterial surfaces [87, 88]. Similarly, polyphenolic components such as tannic acid and derivatives of lignin can cause oxidative stress and disruption of bacterial envelope integrity by means of redox activity [89, 90]. The primary advantage of contact-killing coatings is their localized action and lack of leaching, ensuring long-term stability and minimal environmental release of biocides [91]. However, the accumulation of dead cell debris can hinder performance over the long term, and this approach often needs to be combined with foul-release or self-cleaning designs [92].

#### Release-killing Antifouling

Release-killing coatings function by the controlled release of biocidal agents to maintain long-lasting antimicrobial activity over a certain period (Fig. 2f). Biomass components serve as an excellent platform for encapsulation and sustained release of antimicrobial compounds such as silver nanoparticles [93–95], and plant polyphenols [89]. The active species in such coatings gradually diffuse into the environment, interfering with bacterial physiological processes such as microbial metabolism, quorum sensing, and biofilm maturation [96]. The rate of release can be modulated by adjusting crosslinking density, porosity, or through environmental stimuli such as pH, ionic strength, and temperature [97]. For example, cyclodextrin hydrogels serve as excellent carriers for biocides, where the molecular cavities enable gradual and sustained release of active agents, ensuring long-term antimicrobial efficacy with good biocompatibility [98, 99].

In general, biomass-based coatings achieve antifouling by combining anti-adhesion and biocidal mechanisms. While anti-fouling strategies prevent the initial attachment of proteins and bacteria, biofouling is a progressive process involving a broader hierarchy of foulants. Beyond microfouling, coatings must also resist the settlement of microscopic eukaryotes such as diatoms and algal spores, as well as macroscopic organisms including barnacles and mussels. Furthermore, environmental foulants like silt and mineral sediments can accumulate on surfaces, requiring specific designs such as the antisediment properties found in zwitterionic systems. The integration of these multi-target effects ensures durable and environmentally friendly antifouling performance against complex biological and inorganic threats. The next section introduces synthesis methods used to construct biomass-based coatings with such functional properties.

## Synthesis Methods of Biomass-based Antifouling Coatings

### Chemical Crosslinking

As previously stated, biomass-derived materials are rich in reactive functional groups such as –OH, –NH_2,_ and –COOH, which enable extensive chemical modification. Cross-linking can be achieved through various coupling or grafting strategies using agents such as silane, isocyanates, glutaraldehyde, or epoxy compounds (Table 1). These reactions promote the formation of covalent linkages between biopolymer chains, generating a tunable three-dimensional network. The resulting structure enhances the coating’s mechanical strength, solvent resistance, adhesion, and thermal stability, ultimately extending the service life of antifouling coatings. Several crosslinking approaches have proven effective for biomass-based antifouling systems, including:

#### Carbodiimide-Mediated (EDC/NHS) Crosslinking

**Fig. 3:**
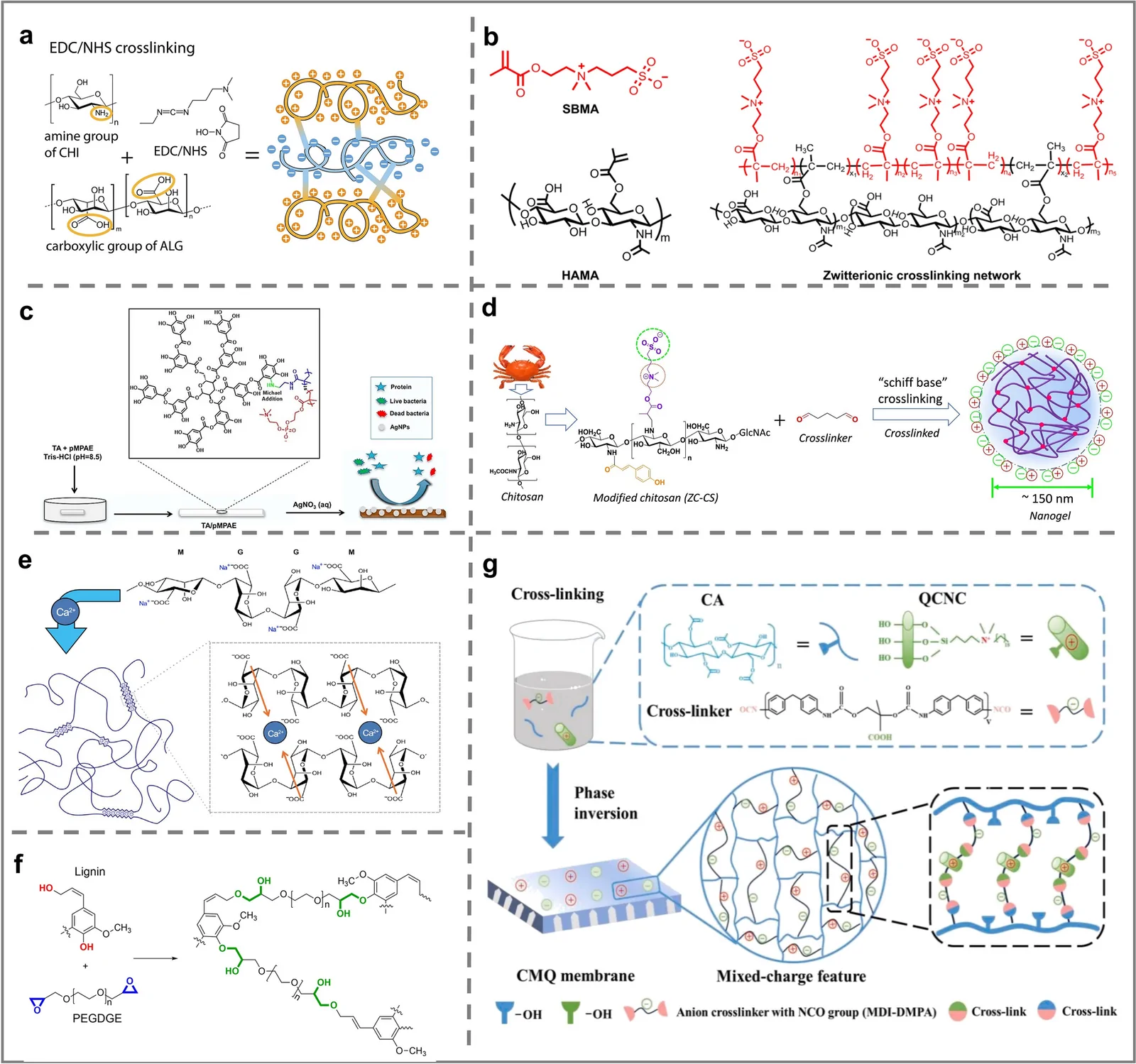
Schematic illustrations of representative chemical crosslinking strategies used in biomass-based antifouling coatings. **a** EDC/NHS-mediated amide coupling. Reproduced with from Ref. [103] under the provisions of the CC BY 4.0 License.** b** Free-radical polymerization of zwitterionic networks. Reproduced with permission from Ref. [104]. Copyright 2022, American Chemical Society. **c** Michael-addition crosslinking. Reproduced with permission from Ref. [105]. Copyright 2024, American Chemical Society. **d** Dynamic Schiff-base formation. Reproduced with permission from Ref. [106]. Copyright 2024, Elsevier. **e** Ca^2+^-induced alginate “egg-box” coordination. Reproduced from Ref. [107] under the provisions of the CC BY 4.0 License.** f** Epoxy crosslinking between lignin and PEGDGE. Reproduced from Ref. [108] under the provisions of the CC BY 4.0 License. **g** Isocyanate (MDI-DMPA) crosslinking yielding mixed-charge cellulose membranes. Reproduced with permission from Ref. [109]. Copyright 2024, Elsevier

The EDC/NHS coupling strategy facilitates the formation of biopolymer networks by promoting reactions between –COOH and –NH_2_ groups to yield stable amide bonds (CO–NH–) with high selectivity [100]. The method is characterized for its excellent solubility in aqueous media, rendering it well-suited for crosslinking aqueous biomaterials [101]. Such materials include carboxyl-containing biomass components (e.g., alginate, oxidized cellulose, carboxylated lignin) and amine-containing polymers (e.g., chitosan, poly (L-lysine)). The resulting stable network structure exhibits excellent antifouling properties. For instance, Zhang et al. utilized an EDC/NHS-mediated coupling reaction to crosslink 2-aminoisonicotinic acid onto chitosan, thereby introducing 2-aminopyridine residues with effective hydration properties, which enhanced the water solubility and antibacterial activity of the latter [102]. In a similar manner, Hautmann et al. utilized this cross-linking method in combination with layer-by-layer self-assembly technology to cross-link the two polysaccharides of negatively charged alginate and positively charged chitosan (Fig. 3a) [103]. The resulting crosslinked polysaccharide network exhibited strong antibacterial performance, highlighting its potential for antifouling applications.

#### Free Radical Polymerization Crosslinking

Free radical polymerization has been demonstrated to be an effective method for the generation of biomass-based antifouling networks, with the formation of carbon–carbon bonds being a key feature of this process. Free radical polymerization is a chain polymerization reaction in which initial free radicals are generated by heat, light, or chemical initiators (such as peroxides and azo compounds). These radicals subsequently react with monomer molecules, resulting in the formation of new radicals and the extension of the polymer chain. This method has been shown to be effective in the polymerization of vinyl functionalized biomass materials, including methyl methacrylate cellulose [110], acrylate chitosan [111], and methyl methacrylate modified carboxybetaine [112]. Jeong et al. prepared poly(methacrylamide carboxybetaine-trifluoroethyl methacrylate) (poly(CBMA-r-TFEMA)) amphiphilic copolymers by free radical polymerization at 70 °C using azobisisobutyronitrile (AIBN) as an initiator, and dopamine to assist in coating application on various substrates, such as stainless steel, showing excellent antifouling properties against marine diatoms and sediment [113]. In another study, Zhang et al. utilized benzophenone (BP) as an initiator to polymerize sulfobetaine methacrylate (SBMA) with methacrylated hyaluronic acid (HAMA) under ultraviolet light (Fig. 3b) [104], thereby forming a hydrogel coating with an interpenetrating network structure on a polymer substrate (including polydimethylsiloxane (PDMS), polyvinyl chloride (PVC), and latex). The coatings markedly reduced protein adsorption (12% vs. 76% on bare PDMS) and inhibited bacterial adhesion. In addition, they exhibited bactericidal activity, achieving killing efficiencies of 85% for *Escherichia coli* and 94% for *Staphylococcus aureus*, as determined by live/dead staining.

#### Michael Addition Crosslinking

Michael addition reaction has emerged as an efficient and versatile cross-linking strategy for the construction of biomass-based antifouling coatings [114, 115]. This reaction involves the nucleophilic addition of thiol (–SH) or –NH_2_ groups to *α*,*β*-unsaturated compounds, forming stable thioether (–C–S–C–) or amine (–C–NH–C–) linkages. Notably, it proceeds under mild aqueous conditions without the need for metal catalysts or external stimuli, making it particularly suitable for preserving the functionality of sensitive biomass-derived components. Thiol-functionalized biopolymers, such as chitosan or gelatin, can readily react with acrylate or maleimide derivatives to form robust and surface-functionalized cross-linked networks [116]. Imbia et al. utilized naturally derived tannic acid as both an adhesive and a Michael acceptor to construct covalent coatings via Michael addition with amino-containing phosphorylcholine copolymers (Fig. 3c) [105]. The coating significantly reduced Bovine serum albumin (BSA) adsorption (from 2.9 to 0.21 μg cm^−2^), demonstrating that Michael addition can effectively enhance the stability and antifouling capability of biomass-based coatings.

#### Schiff Base Crosslinking

Schiff base chemistry enables the formation of imine (C=N) and secondary amine (R–NH–) bonds between aldehyde (–CHO) and amino (–NH_2_) groups, providing stable chemical cross-linking for biomass-based antifouling systems [117–119]. Glutaraldehyde, with two aldehyde groups, is commonly used to crosslink amino-rich biomaterials such as chitosan and proteins, forming networks with tunable properties [120, 121]. For example, Basak et al. prepared a sprayable zwitterion-modified chitosan-based Schiff base crosslinked nanogel (ZC-CSNG) using glutaraldehyde to form Schiff base bonds with chitosan (Fig. 3d) [106]. The Schiff-base-induced network provided structural stability, while the zwitterionic modification markedly reduced BSA adsorption, yielding improved antifouling performance.

Alternatively, aldehyde groups can be introduced by oxidizing natural polysaccharides. Yi et al. oxidized carboxymethyl cellulose to obtain dialdehyde carboxymethyl cellulose (DCMC) for collagen crosslinking. This approach eliminates the toxicity of conventional crosslinkers while enhancing porosity, thermal stability, and biodegradability, providing a greener crosslinking strategy [122]. A key advantage of Schiff base chemistry is the dynamic reversibility of C=N bonds under certain conditions, enabling self-healing in antifouling coatings [123]. Deng et al. developed a hydrogel made from chitosan methacrylate (CSMA) and dialdehyde bacterial cellulose (DABC) [124], where Schiff base bonds formed the initial dynamic network, which was then reinforced through photo-crosslinking. This hydrogel can self-repair by reforming C=N bonds, demonstrating excellent compatibility for long-term biomedical or antifouling applications.

#### Metal Ion Crosslinking

In metal ion crosslinking, positively charged multivalent metal cations (e.g., Ca^2+^, Fe^3+^, Al^3+^, and Zn^2+^) form reversible or partially reversible coordination bonds with negatively charged carboxylates (–COO^−^), phenolic hydroxyl groups (–ArOH), or hydroxyl groups (–OH) in biomass-derived polymers. The alginate-based system is a classic example of this process [125]. As illustrated in Fig. 3e [107], the divalent metal ions are surrounded by the *α*-L-guluronic acid blocks of alginate, forming an “egg-box” structure that significantly enhances the mechanical properties of the coating. For instance, Xiong et al. created a double network hydrogel using alginate and Ca^2+^ coordination [126]. This resulted in high-strength ionic crosslinking, giving the material excellent mechanical properties (with a tensile strength of up to 17.23 MPa) and significantly improving its structural stability in a marine environment. Metal coordination can also form a dense network structure similar to a “metal–organic framework” with polyphenols (such as tannic acid) [127–129], which improves scratch and wear resistance. Furthermore, metal ion cross-linked coatings can respond to external stimuli such as pH value and ionic strength to undergo structural reorganization [130], creating a smart, self-healing, antifouling surface.

#### Epoxy Crosslinking

Epoxy crosslinking involves epoxy groups reacting with nucleophilic functional groups in biomass, such as –NH_2_, –OH, and –SH. These reactions yield secondary amine linkages (–C–NH–C–) with amines and ether or thioether linkages (–C–O–C– or –C–S–C–) with hydroxyl and thiol groups. This chemical reaction is frequently employed for the modification of hydroxyl-rich biomass-derived materials [131], including lignin (Fig. 3f) and gelatin [132, 133]. Rashedi et al. conducted a study in which they crosslinked kraft lignin and starch through PEGDGE under alkaline conditions, thereby forming a covalently linked starch-lignin composite material [134]. This crosslinked structure has been demonstrated to enhance the thermal stability and surface hydrophilicity of starch. Trinh et al. developed a lignin-based hydrogel crosslinked with PEGDGE, where the epoxy-mediated network markedly enhanced mechanical stability (tensile strength up to 80 kPa) while retaining high water content (90%) [135].

In order to enhance the reaction efficiency of epoxy ring opening, Teng et al. introduced primary amine groups into lignin via a Mannich reaction and subsequently crosslinked it efficiently with PEGDGE to fabricate hydrogels with a high lignin content (approximately 71 wt%) [136]. The resulting hydrogels exhibited significantly enhanced mechanical properties, with a shear strength reaching 80 kPa and a storage modulus (G′) of up to 12.7 kPa. Furthermore, epoxy cross-linked biomass coatings have been demonstrated to enhance the adhesion of the coating to the substrate [137], thereby providing a solution to the problem of facile detachment of the coating in antifouling applications.

#### Isocyanate Crosslinking

Isocyanate crosslinking is defined as a reaction between isocyanate (–NCO) groups and –OH or –NH_2_ groups in biomass-derived materials, forming carbamate (–NH–COO–) or urea (–NH–CO–NH–) bonds. Isocyanate compounds are extensively utilized in the modification of biomass materials, with isophorone diisocyanate (IPDI), toluene diisocyanate (TDI), methylene diphenyl diisocyanate (MDI), and hexamethylene diisocyanate (HDI) being prominent examples. Isocyanates have been found to be particularly suitable for crosslinking lignin to construct structurally stable antifouling coatings [76, 138, 139]. Such coatings have been shown to possess certain swelling resistance and surface energy regulation capabilities, while retaining their inherent antioxidant and UV absorption properties. For instance, Zhao et al. crosslinked hydroxyl-terminated cellulose acetate with cationic nanocellulose (QCNC) through carboxyl-containing isocyanate (MDI-DMPA) to construct a coating film with a mixed charge interface (Fig. 3g) [109]. The membrane has been shown to significantly inhibit protein adsorption, with the BSA adsorption amounts being only 8.9 and 12.7 μg cm^-2^ at 12 and 24 h respectively. These levels are considerably lower than the levels observed for the uncross-linked coatings. This finding indicates that isocyanate cross-linking combined with charge regulation significantly enhances resistance to protein contamination.

### Physical Crosslinking

Given the environmental pollution concerns associated with covalent cross-linkers and metal ions, there is an increasing demand for developing antifouling coatings based on non-covalent physical interactions. Physical cross-linking utilizes non-covalent interactions to construct three-dimensional network structures in biomass-based antifouling coatings. This approach enables the formation of reversible, self-healing architectures with tunable properties, making them particularly suitable for dynamic and environmentally responsive applications.

#### Electrostatic Interactions and Layer-by-Layer Assembly

**Fig. 4:**
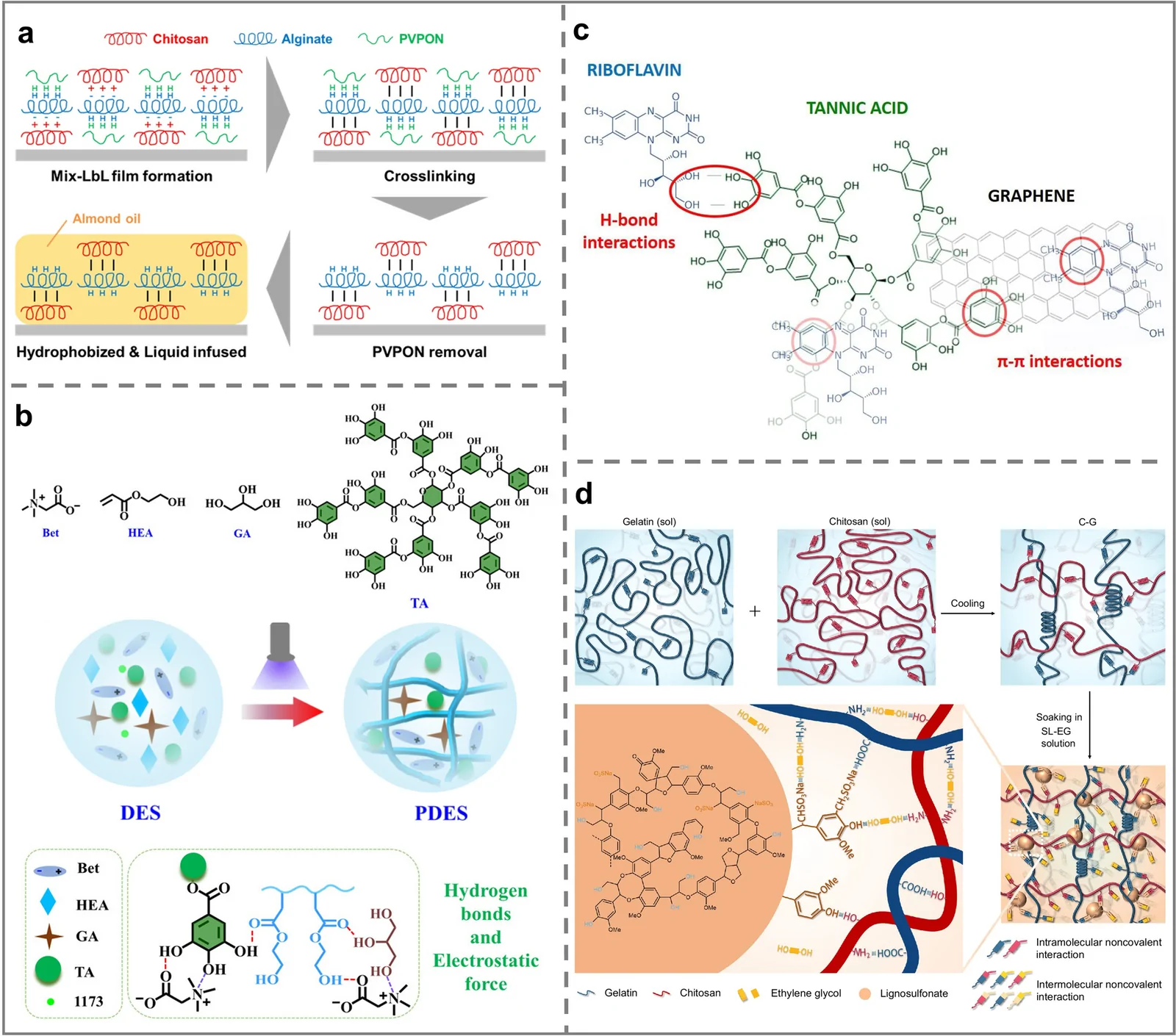
Representative physical crosslinking strategies. **a** Electrostatic interaction: Layer-by-layer (LbL) assembly of oppositely charged chitosan and alginate. Reproduced with permission from Ref. [143]. Copyright 2015, American Chemical Society. **b** Formation of a polymerizable deep eutectic solvent (PDES) network. Reproduced with permission from Ref. [145]. Copyright 2025, American Chemical Society. **c** Tannic acid self-assembles onto graphene nanosheets via π-π stacking. Reproduced from Ref. [146] under the provisions of the CC BY 4.0 License. **d** A lignosulfonate-chitosan–gelatin supramolecular organohydrogel. Reproduced from Ref [147]. under the provisions of the CC BY 4.0 License

Electrostatic interactions between oppositely charged biomass-derived components facilitate the fabrication of antifouling coatings through layer-by-layer (LbL) assembly [140]. By combining cationic species such as chitosan and polylysine with anionic counterparts, including alginate and sulfonated lignin [65, 141, 142], this approach enables the integration of diverse functionalities into a single multilayer platform. The resulting multilayer films exhibit excellent hydration properties, robust resistance to protein adsorption, and responsiveness to environmental stimuli. As shown in Fig. 4a, Manabe et al. utilized electrostatic interactions between chitosan and sodium alginate to fabricate multilayered films via LbL assembly [143]. They demonstrated that film thickness increased linearly with the number of assembly cycles, and the resulting films exhibited robust antibacterial activity and structural stability under artificial seawater conditions. Peng et al. also reported a macroporous sponge dressing based on carboxymethyl chitosan and sodium alginate [144]. Through frozen casting and subsequent acetic acid steam treatment, the electrostatic cross-linking is enhanced, forming a dense and mechanically stable pore-wall structures. Compared with the traditional sol–gel method, this method significantly improves the mechanical strength and still maintains good shearing and compression properties after hydration and expansion.

In addition, the LbL assembly strategy can accurately control the surface microstructure and can be used to prepare bionic anti-fouling coatings. Zhao et al. prepared a nano-layer structure of polyethylene imide derivatives and sodium alginate through LbL assembly [141]. They encapsulated capsaicin in chitosan nanocapsules and fixed the latter in a multi-layer structure to build a bionic surface with coordinated anti-fouling function. The obtained coating showed excellent anti-bacterial adhesion (inhibition rate of 99.2%) and anti-diatom adhesion (inhibition rate of 99.92%), highlighting the effectiveness of the comprehensive strategy in enhancing the anti-fouling performance.

#### Hydrogen Bonding Interactions

Hydrogen bond is a key physical cross-linking mechanism based on the reversible interaction between hydrogen donors and receptors. Biomass-derived components, including chitosan, cellulose, tannic acid, and lignin, contain rich polar groups, such as –OH, –NH_2_, and –COOH, which can form a wide range of hydrogen bonds inside and between the polymer chain. These interactions promote the formation of dynamic three-dimensional networks, thus improving film formation, mechanical strength, and self-healing ability. Lin et al. designed an anti-fouling coating based on tannin (TA) and bottle brush polymer (BBP) co-deposition [148]. This coating does not need the introduction of covalent crosslinkers but forms a stable physical network through multiple hydrogen bonds. BBP consists of a polyurethane (PU) backbone and polyethylene glycol (PEG) side chains that can form multiple hydrogen bonds with the phenolic hydroxyl groups in TA. Molecular dynamics simulations and characterization methods such as 2D IR and ^1^H NMR confirmed that the PU main chain and PEG side chains can both strongly hydrogen bond with TA, achieving a maximum binding energy of 86.3 kJ mol^-1^. Compared with linear polymers, the branched structure of BBP helps form a denser network, thereby improving the stability of the coating.

Recently, deep eutectic solvents (DES), which rely on hydrogen bonding networks, have gained attention in coating design. DES are formed by mixing hydrogen bond donors and acceptors to create low-melting point mixtures with environmentally friendly and tunable properties [149]. Zhang et al. proposed a polymerizable DES system based on betaine and TA [145], incorporating hydroxyethyl acrylate (HEA) and glycerol (GA) (Fig. 4b). Under UV initiation, this system formed a dense hydrogen-bonded network with shear strength up to 689.5 kPa and tensile strength reaching 197.88 MPa. These properties indicate that polymerizable DES platforms may offer useful design elements for durable antifouling coatings, particularly where mechanical robustness and aqueous stability are required.

#### π-π Interactions

Π-π stacking plays a crucial role in constructing highly stable biomass-based antifouling coatings. Biomass-derived components rich in aromatic structures, such as lignin and tannic acid, can leverage their π-electron systems to form strong non-covalent interactions with aromatic nanofiller materials (such as CNTs and graphene) (Fig. 4c) [146], thereby constructing enhanced physical crosslinking networks. He et al. utilized the π-π stacking interactions between the aromatic ring structure of TA and graphene to establish a stable dispersion interface [150]. This strategy markedly improved graphene’s dispersion and interfacial compatibility within the polymer matrix, preventing performance degradation caused by agglomeration. Tan et al. functionalized multi-walled carbon nanotubes (MWCNTs) with photoactive tannic acid via π-π stacking and incorporated them into acrylated epoxidized soybean oil (AESO) coatings [151]. At 0.8 wt% loading, tensile strength and modulus increased by 109% and 409%, demonstrating effective reinforcement through improved dispersion and interfacial interaction.

#### Supramolecular Interactions

Supramolecular interaction refers to the structured organization of molecular systems maintained by non-covalent forces (such as hydrogen bonds, electrostatic attraction and π-π stacking) [152]. Compared with covalent cross-linking, supramolecular interaction shows better selectivity, environmental responsiveness, and inherent self-healing ability, so that it can accurately regulate the surface performance and anti-fouling mechanism of the coating at the molecular level. Gu et al. developed a supramolecular hydrogel adhesive based on chitosan, sodium lignin sulfonate, and gelatin [147]. As shown in Fig. 4d, their design completely bypassed the use of a covalent cross-linking agent, instead relying on a supramolecular interaction network, comprising electrostatic pairing, hydrogen bonding between positively charged chitosan and negatively charged sodium lignin sulfonate, and π-π stacking interactions driven by the aromatic groups in sodium lignin sulfonate. These synergistic interactions together form a uniform and stable network.

Based on this concept, Huo et al. introduced peptide-polyphenol coatings with adjustable assembly kinetics [153]. In this system, TA interacts with cationic peptides through electrostatic attraction, while citric acid regulates the assembly by forming multiple hydrogen bonds with TA and peptides. The resulting coatings showed reduced protein adhesion, illustrating that controlled supramolecular interactions can be used to construct multifunctional anti protein adsorption surfaces.

Compared with chemical methods, physical cross-linking methods have advantages in reversibility, dynamic behavior and environmental responsiveness, but their long-term stability often faces challenges. Hybrid strategies combining physical and chemical crosslinking mechanisms frequently provide optimal performance in practical biomass-based antifouling applications.

In summary, while diverse synthesis methods provide a versatile toolkit for constructing biomass-based coatings, the crosslinking degree emerges as a decisive parameter in modulating the delicate balance between mechanical integrity and antifouling efficacy. A higher crosslinking density generally reinforces the mechanical robustness of the coating by establishing a rigid and interconnected polymer network, which enhances properties such as hardness, Young’s modulus, and resistance to hydrodynamic shear. However, a “tipping point” exists where excessive crosslinking begins to compromise antifouling performance. For passive strategies, dense networks reduce the free volume within the matrix and restrict the conformational mobility or swelling potential of hydrophilic segments such as polysaccharides or betaines, thereby hindering the formation of a robust hydration layer. For active strategies, high crosslinking can increase steric hindrance and diffusion resistance, which limits the contact-accessibility of antimicrobial moieties or slows the controlled release of encapsulated biocides. Consequently, identifying an optimal “synergistic window” for the crosslinking degree is essential to ensure that biomass-based coatings maintain structural durability without sacrificing the interfacial dynamics required for long-term fouling resistance.

## Biomass Materials Used in Antifouling Coatings

The selection of biomass materials for antifouling coatings is fundamentally driven by their diverse chemical structures and intrinsic biological properties [38, 154]. These materials can be categorized based on their primary roles in countering biofouling: polysaccharides and proteins are predominantly utilized for their abundant hydrophilic functional groups to construct passive hydration barriers, while tannins and lignin utilize their polyphenolic architectures to provide both antioxidant and active biocidal activities. Furthermore, the molecular versatility of these precursors allows for tailored chemical modifications, such as the introduction of zwitterionic betaine moieties or cationic charges, to achieve targeted repulsion or contact killing functions. By aligning these intrinsic properties with specific antifouling requirements, biomass-based systems offer a customizable platform for sustainable surface protection. The following subsections detail the characteristics, modification strategies, and performance of these major biomass categories.

### Lignin

**Fig. 5:**
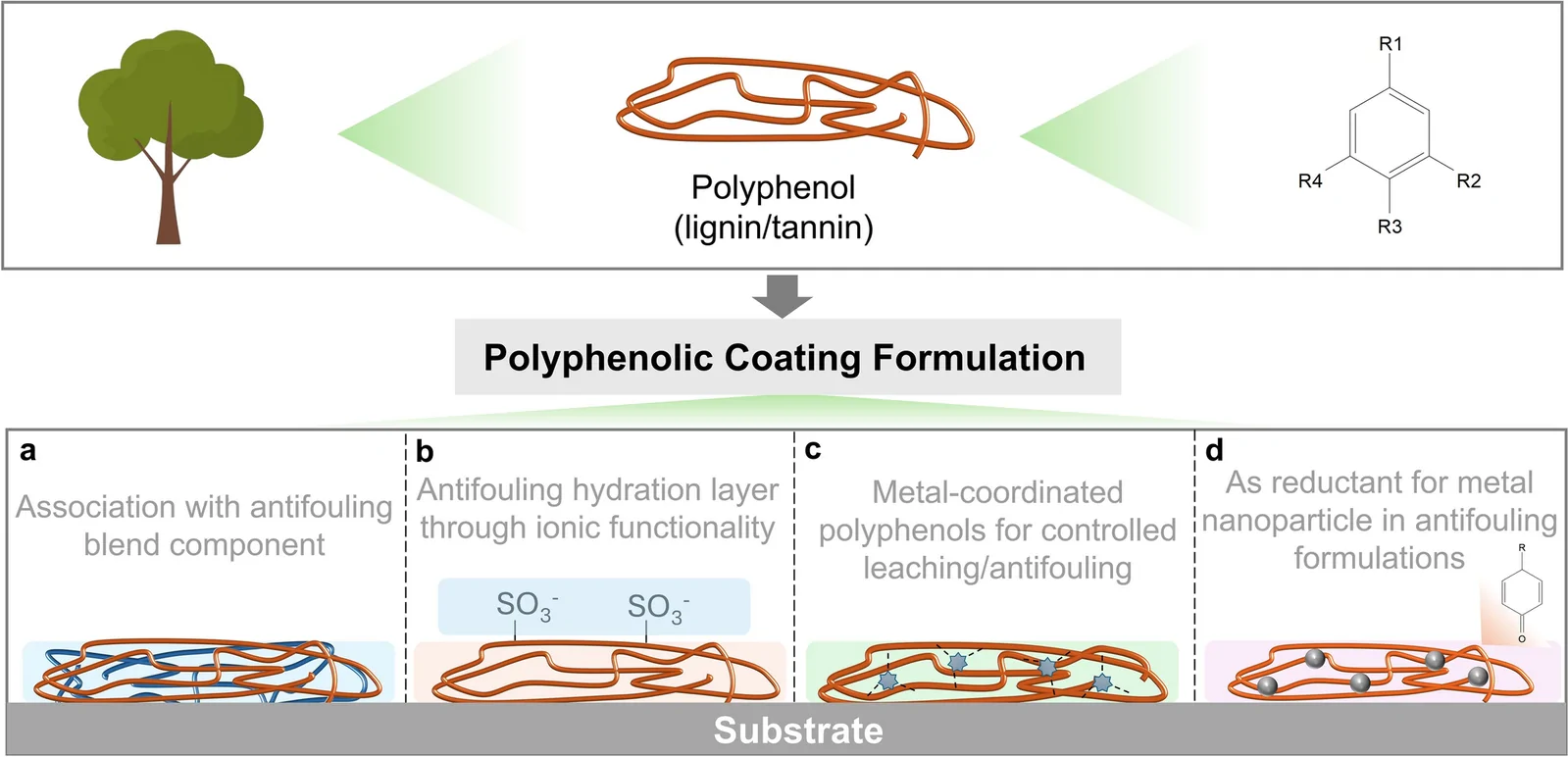
Schematic illustration of polyphenolic coating **a** Association with antifouling blend component. **b** Antifouling hydration layer through ionic functionality. **c** Metal-coordinated polyphenols for controlled leaching/antifouling. **d** As reductant for metal nanoparticle in antifouling formulations

Coatings with antifouling properties relying on the hydrophobic and polymeric properties of lignin have been exemplified in formulations combining acetic acid lignin and poly *N*, *N*-dimethylacrylamide (PDMA) [155]. This approach utilizes the amphiphilic association between hydrophobic lignin and hydrophilic PDMA to achieve a balanced blend interface (Fig. 5a). The coating precursor solution used dimethyl formamide (DMF) to dissolve the two polymeric components: hydrophobic lignin and hydrophilic PDMA. Without any covalent cross-linker, the swelling properties and stability of the produced coating were completely sustained by chain-entanglement (noncovalent cross-linking) and secondary forces (e.g., π-π) for lignin-driven hydrophobic crosslinking with the bottom substrate. In this case, the choice of solvent for proper dispersion between the lignin and PDMA can be expected to be crucial for the properties in the dried coating formulation. This is because DMF is considered a strong lignin solvent [156], and more specifically, it reduces the driving force for aromatic self-assembly [157], also occurring in dissolved lignin systems [158], allowing improved dispersion between the two polymers prior to solution casting and drying. The hydrophilic top-layer (PDMA-dominated) ensured antifouling properties by a strong hydration layer, whereas acetic acid lignin can be considered one of the more hydrophobic lignin sources and offers adhesion to various substrates.

Water-soluble lignins with anionic character can be derived from, for example, sulfonated Kraft lignin. By incorporating these ionic functionalities (Fig. 5b), lignin-based coatings can construct passive antifouling hydration layers to mitigate the adsorption of hydrophobic foulants [159]. Kraft lignin has also been exploited as a component in antifouling formulations resisting fouling by polar components. More specifically, methacrylated Kraft lignin was chemically (covalent and by secondary interactions) and physically cross-linked in a matrix with poly(sulfobetaine methacrylate) (PSBMA) and cellulose nanofibrils (CNFs) [160]. The improved antifouling behavior toward anionic and cationic proteins aqueously dispersed was attributed to the strong water hydration layer formed by the anionic components in the formulation. An increasing lignin content, however, reduced the strength and extension of this shielding water layer due to its hydrophobic nature.

Other strategies involve the use of metal-coordinated polyphenols to achieve controlled leaching and sustained antifouling properties (Fig. 5c). Additionally, lignin can serve as a reductant for metal nanoparticles in antifouling formulations (Fig. 5d) [161]. In this case, lignin undergoes oxidation into its quinone forms, stabilizing the AgNPs, ensuring high bactericidal efficacy. As such, the potential of lignin in antifouling applications is extensive, but requires careful consideration of functional groups and macromolecular properties, thus affecting their performance and suitability toward the specific application.

### Tannin

Similar to lignins, tannins are a heterogeneous group of polymeric phenolic compounds present in plants. Commonly, these polyphenols are separated into hydrolysable and condensed tannins, with molecular weights ranging between 500 and 3000 Da [168], making this class structurally smaller than what is the general case for lignins. Meanwhile, their hydrophilicity and rich content of oxygen functionalities make them prone to form strong complexes with minerals or other biopolymers, making them an interesting option as a component during the synthesis of antifouling coatings (Table 2). Employing antifouling tannins of relatively low molecular weight, and without sufficient steric entrapment in the coating, or any covalent cross-linking causes leaching when exposed to experimental conditions like marine environments [169, 170], however, by controlling the leaching rate of tannin-based pigments complexed (e.g., cupric tannate) with anti-fouling agents in marine paints have been proven effective in controlling growth of fouling marine species [171, 172], as has the leachate of non-toxic (non-copper based) tannin formulations [173]. Efforts have also been made in order to reduce the solubility of antifouling tannins without using metals, more precisely by using high temperature conditions in combination with alcohols and acid for subsequent pigment synthesis by adsorption on activated carbon, and then applied on steel panels [174]. After application and long-term submersion, the tannin-based coatings perform similarly to of commercial coatings in terms of anti-fouling, with residual tannins remaining after 7 months, indicating that the tannins, to a certain extent, are retained and not completely leached out of the painting. Other strategies that completely avoid the issue with leaching involve cross-coupling of, for example, tannic acid and (3-Chloropropyl) trimethyoxysilane (CTS) with polyethyleneimine (PEI) under alkaline conditions in efforts to reduce fouling of membranes by organic contaminants [175]. Benefiting from the high hydrophilicity of tannic acid, oil rejection and organic fouling were improved. Similar effects have been achieved by metal coordination of tannic acid and PEI [176].

### Betaine

#### Sulfobetaine

**Fig. 6:**
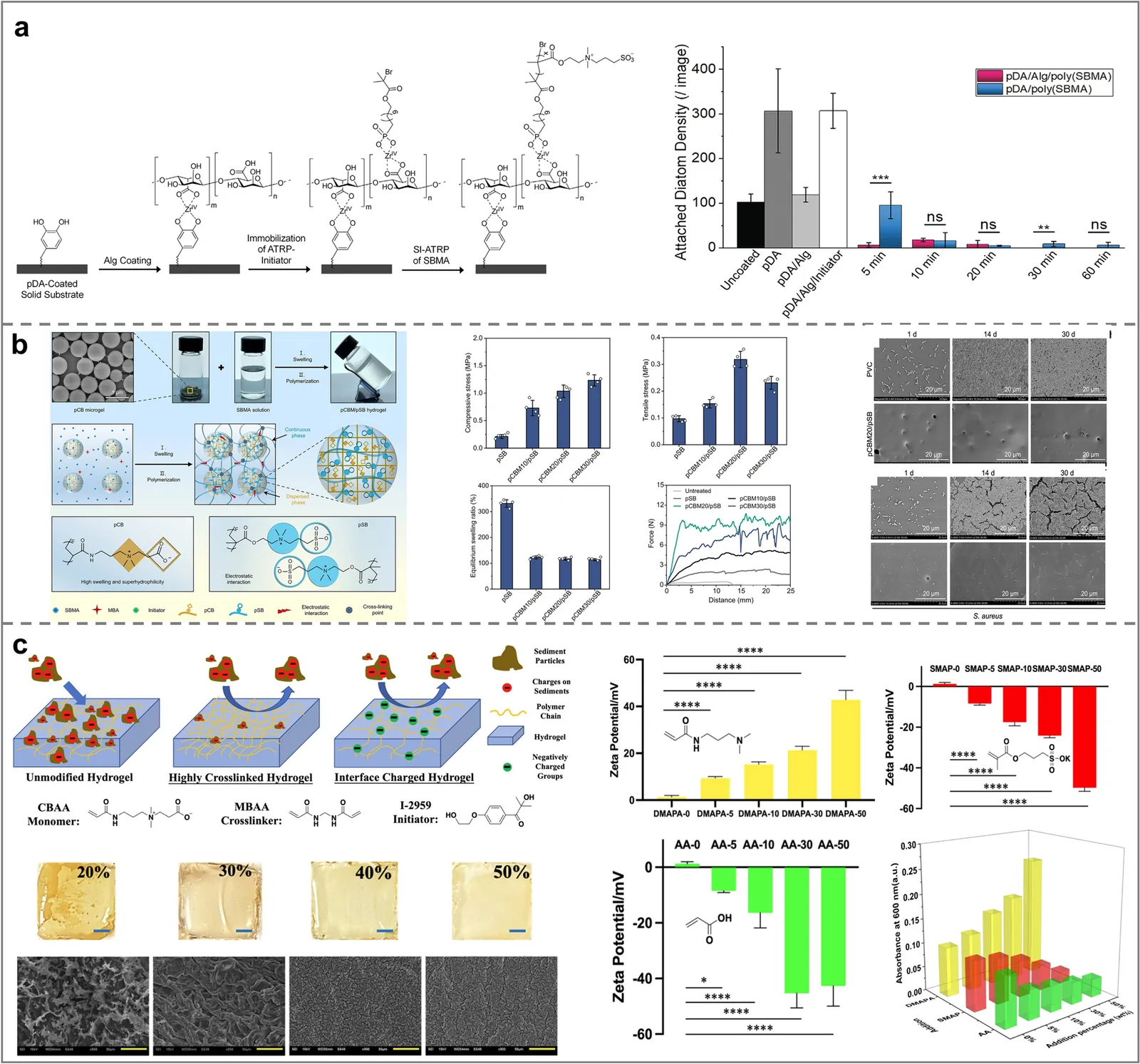
Representative zwitterionic antifouling strategies. **a** Schematic of poly(SBMA) brush formation and its effect on diatom adhesion. Reproduced with permission from Ref. [181]. Copyright 2024, Wiley.** b** Zwitterionic pCBM-reinforced pSB network and its mechanical, interfacial, and antifouling performance. Reproduced from Ref. [182] under the provisions of the CC BY 4.0 License. **c** Antisediment design of PCBAA hydrogels, including surface charge regulation, morphology, and mud-adhesion behavior. Reproduced with permission from Ref. [183]. Copyright 2024, American Chemical Society

Sulfobetaine (SB) zwitterionic polymers have been extensively utilized in antifouling coatings due to their surface charge neutrality and strong hydration capacity [177], as summarized in Table 3. However, SB polymers typically exhibit low mechanical strength and adhesion, and are susceptible to cracking, peeling and wear when utilized as coating materials in isolation. To enhance the durability of SB-based coatings, researchers have investigated methodologies such as copolymerization [178], multilayer structures [179] and hybrid material integration [180]. Lee et al. developed a multilayered alginate/poly(SBMA) coating (Fig. 6a) [181]. The outer SBMA layer contributed to a dense hydration barrier, while the alginate matrix improved surface adhesion, thereby enhancing biofouling resistance while maintaining structural integrity. Their study demonstrated that this hybrid design reduced marine diatom adhesion by 94% compared to alginate-only coatings, highlighting the synergistic effect of natural polysaccharides and zwitterionic hydration layers. A microgel-reinforced zwitterionic strategy was developed in which poly(carboxybetaine) microgels (pCBM) are incorporated into a poly(sulfobetaine) (pSB) network to overcome the mechanical fragility of pure SB hydrogels (Fig. 6b) [182]. The interpenetrating pCBM/pSB architecture dissipates stress effectively, enhancing both mechanical robustness and interfacial adhesion. This reinforced network maintains integrity under shear, bending and abrasion, while providing long-term resistance to *E. coli* and *S. aureus* adhesion and biofilm formation. The microgel-reinforcement concept thus offers a durable and strongly adherent design pathway for zwitterionic SB-based antifouling coatings.

#### Carboxybetaine

Carboxybetaine (CB) represents another important class of zwitterionic materials that have shown remarkable antifouling properties in various coating applications [184]. Unlike SB, CB contains a carboxylate anion group paired with a quaternary ammonium cation, creating a strong dipole moment while maintaining overall charge neutrality. This unique structure enables CB to form a robust hydration layer through ionic solvation effects, which serves as a physical and energetic barrier against fouling organisms and proteins. Song et al. systematically optimized poly(carboxybetaine acrylamide) (PCBAA) hydrogels to enhance antisediment performance in marine environments [183]. As shown in Fig. 6c, two optimization strategies were applied: (i) increasing crosslinking density to reduce pore size and (ii) introducing charged comonomers to regulate interfacial electrostatics. Increasing solid content resulted in a denser network structure, while zeta potential measurements confirmed that negatively charged comonomers such as AA and SMAP effectively minimized sediment adhesion. Overall, these structural and interfacial optimizations enabled PCBAA hydrogels to achieve improved antisediment performance without compromising antifouling properties.

### Polysaccharides

#### Chitosan

Chitosan is a natural polysaccharide primarily extracted from the shells of crustaceans (such as shrimp and crab) through deacetylation treatment. Compared to other biomass-based materials, chitosan has gained widespread application in antifouling coatings, primarily due to its amino functional group (–NH_2_), which confers three distinct advantages:

Intrinsic antibacterial ability: Chitosan has broad-spectrum antibacterial properties. Its antibacterial mechanism is mainly based on the cationic properties of amino groups (–NH_3_^+^), which interact electrostatically with negatively charged components on the bacterial cell membrane, thereby destroying the integrity and permeability of the membrane, leading to cell death and inhibiting the formation of biofilms [196]. However, there are limitations to using chitosan alone as antibacterial coatings, such as the accumulation of cell fragments, which promotes biofilm regeneration. Therefore, recent studies tend to use chitosan in combination with other functional components to enhance its long-term antibacterial properties.

**Fig. 7:**
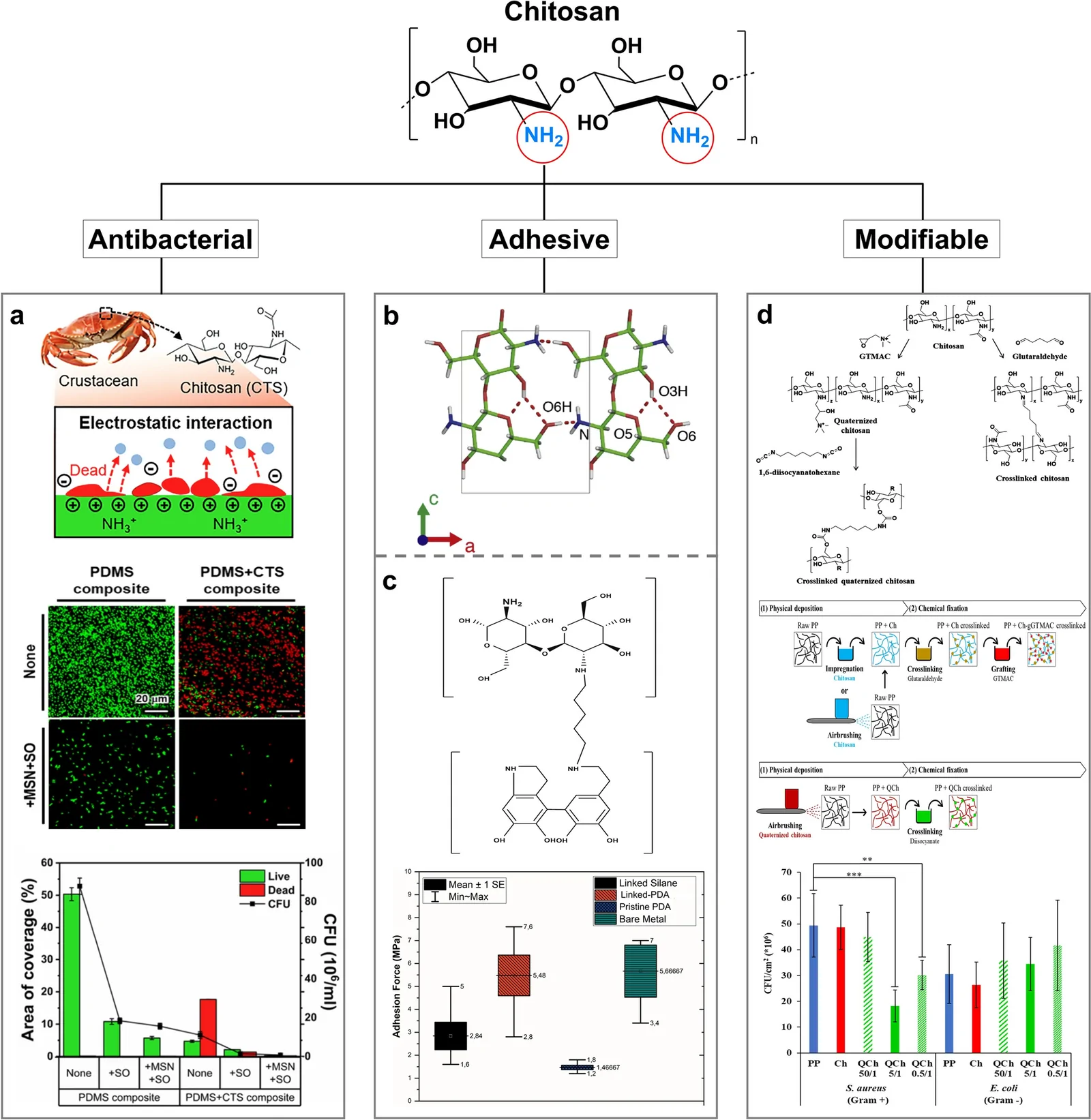
Multifunctionality of chitosan. Antibacterial: **a** Schematic of chitosan-based antibacterial mechanism, live/dead staining of bacteria on PDMS and PDMS + CS composites with or without MSN + SO, and corresponding quantitative analysis of bacterial coverage and CFU count. Reproduced with permission from Ref. [197]. Copyright 2024, American Chemical Society. **b** Molecular model illustrates hydrogen bonding between chitosan chains. Reproduced with permission from Ref. [198]. Copyright 2018, Elsevier. **c** Schematic of the glutaraldehyde-linked PDA-chitosan coating and adhesion strengths of chitosan coatings on different interfacial layers. Reproduced from Ref. [199] under the provisions of the CC BY 4.0 License. **d** Synthetic routes for crosslinked and quaternized chitosan, their deposition and chemical fixation onto PP substrates, and antibacterial performance on coated PP fabrics against *E. coli* and *S. aureus*. Reproduced from Ref. [200] under the provisions of CC-BY-NC-ND 4.0

Jang et al. mixed chitosan, silicone oil, and mesoporous silicon dioxide nanoparticles (MSN) into a polydimethylsiloxane (PDMS) matrix to prepare a multifunctional smooth coating [197]. As shown in Fig. 7a, chitosan provides electrostatic antibacterial activity, while the infused oil layer prevents the retention of dead cells, and the MSN reservoir maintains continuous oil release. PDMS–chitosan alone kills bacteria but leads to substantial debris accumulation. With the addition of oil and MSN, surface coverage is markedly reduced, and strong short- and long-term biofilm inhibition is achieved. These results demonstrate that durable antifouling performance requires the combined action of antibacterial and debris-release mechanisms rather than bactericidal activity alone.

**Table 1:** Summary of crosslinking methods, reactive groups, and formed linkages

Crosslinking method	Reactive groups	Formed linkages
EDC/NHS crosslinking	–COOH and –NH_2_	–CO–NH–
Free radical polymerization	–C=C– and –C=C–	–C-C–
Michael addition	*α*,*β*-unsaturated compounds and –SH/–NH_2_	–C–S–C–/–C–NH–C–
Schiff base reaction	–CHO and –NH_2_	–C=N–/–CH_2_–NH-
Metal ion crosslinking	–COOH/–OH and metal ion	Coordination bonds
Epoxy crosslinking	Epoxy groups and –NH_2_/–OH/–SH	–C–O–C–/–C–NH–C–/–C–S–C–
Isocyanate crosslinking	–NCO and –OH/–NH_2_	-NH-COO–/–NH–CO–NH–

Excellent film-forming performance and adhesion: The hydroxyl groups and amino groups in chitosan are excellent hydrogen bond donors and receptors. These functional groups not only improve film-forming ability by enhancing the inter-chain interaction, but also form hydrogen bonds with the polar groups on the surface of the substrate, thus improving adhesion. Recent research on neutron diffraction and density functional theory (DFT) has been used to clarify the key hydrogen bond patterns in the structure of chitosan crystals [198], especially the O6–H⋯N and HO3⋯O5/O6 configurations (Fig. 7b). These hydrogen bonds play a crucial role in enhancing adhesion, especially on substrates rich in polar functional groups, such as polymethyl methacrylate (PMMA) and polyethylene terephthalate (PET), which help to form a stable interface layer.

**Table 2:** Summary of polyphenolic (lignin and tannin) biomass coatings for antifouling applications

Biomass type	Formulation	Antifouling mechanism	Antifouling performance	References
Organosolv lignin	PEG-PU/lignin	Amphiphilic association	Total biomass determination (crystal violet staining and the MTT assay): *Pseudoalteromonas atlantica:* ~ 0.3	[162]
Sodium ligninsulfonate	MXene/METAC/lignin	Hydrophilic layer, contact-killing	Antibacterial rate (reduction of bacteria vs control):	[163]
			*E. coli:* > 90%,	
			*S. aureus:* > 90%;	
			Anti-microalgae:	
			*Porphyridium:* 85.1%	
			*Dunaliella:* 83.5%	
Kraft lignin	Dual aminated lignin	Hydrophilic layer	BSA adsorption: 27.94 ± 1.47 μg cm^−2^	[164]
Tannic acid	MXene/PEIS/TA	Hydrophilic layer, contact-killing	Antibacterial rate:	[165]
			*E. coli:* 96.2%,	
			*S. aureus:* 91%;	
			Anti-microalgae:	
			*Porphyridium:* 99.4%	
			*Dunaliella:* 99.2%	
Tannic acid	TA/ADH/GO/EPUR	Contact-killing	Antibacterial rate:	[166]
			*E. coli:* > 99%,	
			*S. aureus:* > 99%,	
			*P. aeruginosa:* > 99%;	
Tannic acid	OVA/TA	Hydrophilic layer, contact-killing	Adhesion force of bacteria:	[167]
			*E. coli:* 122.86 nN	
			(coated), 766.87 nN (uncoated),	
			*S. aureus:* 225.93 nN	
			(coated); 972.1 nN (uncoated)	

Methacryloxyethyltrimethyl ammonium chloride (METAC), Polyethyleneimine (PEI), Tannic acid (TA), Adipohydrazide (ADH), Graphene oxide (GO), Organosilane-modified epoxy (EPUR), Ovalbumin (OVA)

Beyond hydrogen bonding, the primary amine groups of chitosan provide additional pathways to reinforce interfacial adhesion through covalent interactions. Glutaraldehyde reacts with these amine groups to form Schiff base linkages, generating a stable crosslinked network at the interface. When this reactive chitosan layer is combined with dopamine or polydopamine (PDA) substrates (Fig. 7c) [199], catechol-mediated interactions and secondary bonding further strengthen the interface. This synergistic amine-aldehyde-catechol coupling complements the intrinsic hydrogen bonding capability of chitosan and offers a robust strategy for enhancing the adhesion and durability of chitosan-based coatings on diverse substrates.

Facile chemical modification: The highly reactive activity of amino groups enables chitosan to undergo a variety of chemical modifications. These groups can carry out acylation, alkylization, and Schiff base formation reactions, thus introducing various functional groups, such as quaternary ammonium groups to enhance antibacterial activity, or carboxyl groups to chelate metals. In addition, amino groups can be used as cross-linking sites to react with cross-linking agents such as glutaraldehyde to form a three-dimensional network, thus improving mechanical properties and controlling swelling behavior.

**Table 3:** Summary of mechanical and antifouling performance of betaine-based antifouling coatings

Betaine type	Copolymer component	Mechanical properties	Antifouling performance	References
SBMA	SPI-SBMA	Breaking strain: 66% to 96%	BSA adsorption: 12.63 to 21.25 mg g^−1^ (increased with SPI)	[185]
	MSA/MAC/SBMA	Coefficient (COF): 0.088 to 0.067	BSA adsorption: 799 to 105 ng cm^−2^	[91]
	SBMA-AM/SA-MXene/Ag-PHMB	Tensile stress: 0.1 (SBMA-AM) to 5.80 MPa	no significant BSA observed by a fluorescence microscope	[186]
	Poly(GMA-co-SBMA)	COF: 0.711 to 0.043	BSA adsorption: decreased by 76%	[187]
	AZO-SBMA	Coating density: 0.11 to 0.31 mg cm^–2^	BSA adsorption: decreased by 76%	[188]
	PEI-g-SBMA/TA-Fe^3+^	COF: 0.55 to 0.04	BSA adsorption: 54.39 to 1.34 µg cm^−2^	[189]
	QCS-poly(AA/DMA/SBMA) /TCNCs	Tensile strength: 59.15 to 77.69 kPa	BSA adsorption relative: < 10%	[190]
	P(MSA-co-SBMA-co-MAPTAC)	COF: 0.126 to 0.054	BSA adsorption: decreased by 87.2%	[191]
	BSA@PSBMA	No data	BSA adsorption: 1131 (native BSA-coated sensor) and 2313 (Au sensor) to 50.3 ng cm^−2^	[192]
	SBMA-DA	Compressive stress: 0.56 (pure SBMA) to 4.7 MPa; Tensile fracture stress: 54 to 210 kPa	BSA adsorption: ~ 20 to ~ 120 µg	[193]
	PAM/QCS/SBMA	Tensile strength: 258.3 kPa; Elastic modulus: 44.5 kPa, COF: 0.053	BSA adsorption: △Abs from ~ 0.012 (PVC catheter) to ~ 0.003	[194]
CBMA	PU fabric/CNT/ZCB	Tensile modulus: 66.5 (PU) to ~ 70 MPa (PU/ZCB)	BSA adsorption: fluorescent intensity from 36.5 (PU) to 1.7 (PU/ZCB), decreased by 95.3% fabric	[195]

Soybean protein isolate (SPI), mono-2-(methylpropoxy) ethyl succinate (MSA), methacrylate trimethyl ammonium chloride (MAC), acrylamide (AM), sodium alginate (SA), polyhexamethylene biguanide (PHMB), glycidyl methacrylate (GMA), azobenzene (AZO), poly(ethylenimine) (PEI), Tannic acid (TA), quaternized chitosan (QCS), methacrylate anhydride dopamine (DMA), tunicate cellulose nanocrystals (TCNCs), [3-(methacryloylamino)propyl] trimethylammonium chloride (MAPTAC), dopamine (DA), zwitterionic polyCBAA (ZCB)

Fouilloux et al. developed a coating strategy for inert polypropylene substrates using quaternary ammonium chitosan [200]. They compared two cross-linking methods (Fig. 7d): the traditional cross-linking of glutaraldehyde and the sequential method of quaternary ammonization and then isocyanate cross-linking. The sequential method retains more antibacterial active sites by minimizing the consumption of amino groups, and shows a better antibacterial effect on Staphylococcus aureus at a crosslinking agent ratio of 5:1. This highlights a fundamental design challenge: excessive cross-linking will enhance the network strength but sacrifice biological activity; while insufficient cross-linking will reduce stability. The success of sequential modification shows that separating the functionalization and cross-linking steps can solve this trade-off problem and provide a widely applicable strategy for designing durable and powerful polymer coatings.

Despite these advantages, chitosan exhibits certain limitations, including poor solubility under neutral and alkaline conditions, mechanical brittleness, and limited durability, which restrict its direct application in antifouling coatings. To address these challenges, researchers have developed solutions from multiple perspectives by combining chitosan with other materials through chemical crosslinking or physical interactions to form composite coatings. The specific strategies and their outcomes are detailed in Table 4.

**Table 4:** Chitosan-based antifouling coatings: crosslinking methods, antifouling mechanisms, and antibacterial properties

Coatings	Chitosan interaction methods	Antifouling mechanism	Antibacterial performance	References
CS-ARG/PVA/CUR	Covalent bonding (NHS/EDC crosslinking), Hydrogen bonding	Release-killing antifouling, Contact-killing antifouling	Zone of inhibitions:	[201]
			*E. coli*: 8 mm;	
			*S.aureus*: 15 mm	
CS-GA	Covalent bonding (Michael addition, Schiff base crosslinking), Hydrogen bonding	Contact-killing antifouling	Zone of inhibitions:	[202]
			*E. coli*: 0.65 mm;	
			*S. aureus*: 1.11 mm	
SBMA-CS-pCA	Covalent bonding (Michael Addition, EDC/NHS crosslinking)	Hydration layer formation, Contact-killing antifouling	MIC:	[106]
			*S. aureus*: 1.5 mg mL^−1^;	
			*S. epidermidis*: 0.2 mg mL^−1^	
PDMS-CS-MSN-SO	Hydrogen bonding	Slippery surface antifouling, Contact-killing antifouling	Total bacterial coverage area:	[197]
			*E. coli*: 0.2%;	
			*S.aureus*: 0.2%	
CS NPs modified PEG	Physical adsorption, Hydrogen bonding	Hydration layer formation, Charge repulsion antifouling	Bacterial adhesion:	[203]
			*E. coli*: reduced by 88%	
PEG/CS	Hydrogen bonding	Hydration layer formation, Charge repulsion antifouling	Antibacterial rate (reduction of bacteria vs control):	[204]
			*S. mutans*: 94.16%	
QCS/AAM/SBMA	Hydrogen bonding	Hydration lubrication antifouling, Contact-killing antifouling	Zone of inhibitions:	[194]
			*E. coli*: 4.82 mm;	
			*B. subtilis*: 5.15 mm	
CMC-Ag-PU	Covalent bonding (Michael Addition)	Hydration layer formation, Release-killing antifouling	Zone of inhibitions:	[205]
			*E. coli*: 4.56 mm;	
			*S. aureus*: 6.67 mm	
Cu_2_O/Ag/CS	Covalent bonding (Michael addition, Schiff base crosslinking), Hydrogen bonding	pH-responsive release-killing, Photocatalytic antifouling	MIC:	[206]
			*S. aureus*: 0.1 mg mL^−1^;	
			*Pseudomonas aeruginosa*: 0.1 mg mL^−1^	
p-CMC/PAAc-g-PEBA	Ionic bonding, Coordination bonding, Hydrogen bonding	Hydration layer formation, Contact-killing antifouling	Antibacterial rate:	[207]
			*E. coli*: 99.98%	
CAP/CS/TpPa-1	Hydrogen bonding, Electrostatic interaction	pH-responsive release-killing antifouling, Surface charge repulsion	Antibacterial rate:	[208]
			*S. aureus*: 91.68%;	
			*P. aeruginosa*: 92.84%	
ZnO/CS	Hydrogen bonding	Release-killing antifouling, Contact-killing antifouling	MIC:	[209]
			*E. coli*: 50 µg mL^−1^;	
			*S. aureus*: 25 µg mL^−1^	

Arginine (ARG), Polyvinyl alcohol (PVA), Curcumin (CUR), Gallic acid (GA), p-Coumaric acid (pCA), Minimum inhibitory concentration (MIC), Polydimethylsiloxane (PDMS), Mesoporous silica nanoparticle (MSN), Silicone oil (SO), Polyethylene (PE), Quaternary Ammonium Chitosan (QCS), Acrylamide (AAM), O-carboxymethyl chitosan (CMC), Acrylic Acid (AAc), Polyether-block-amide (PEBA), Capsaicin (CAP)

#### Cellulose

**Fig. 8:**
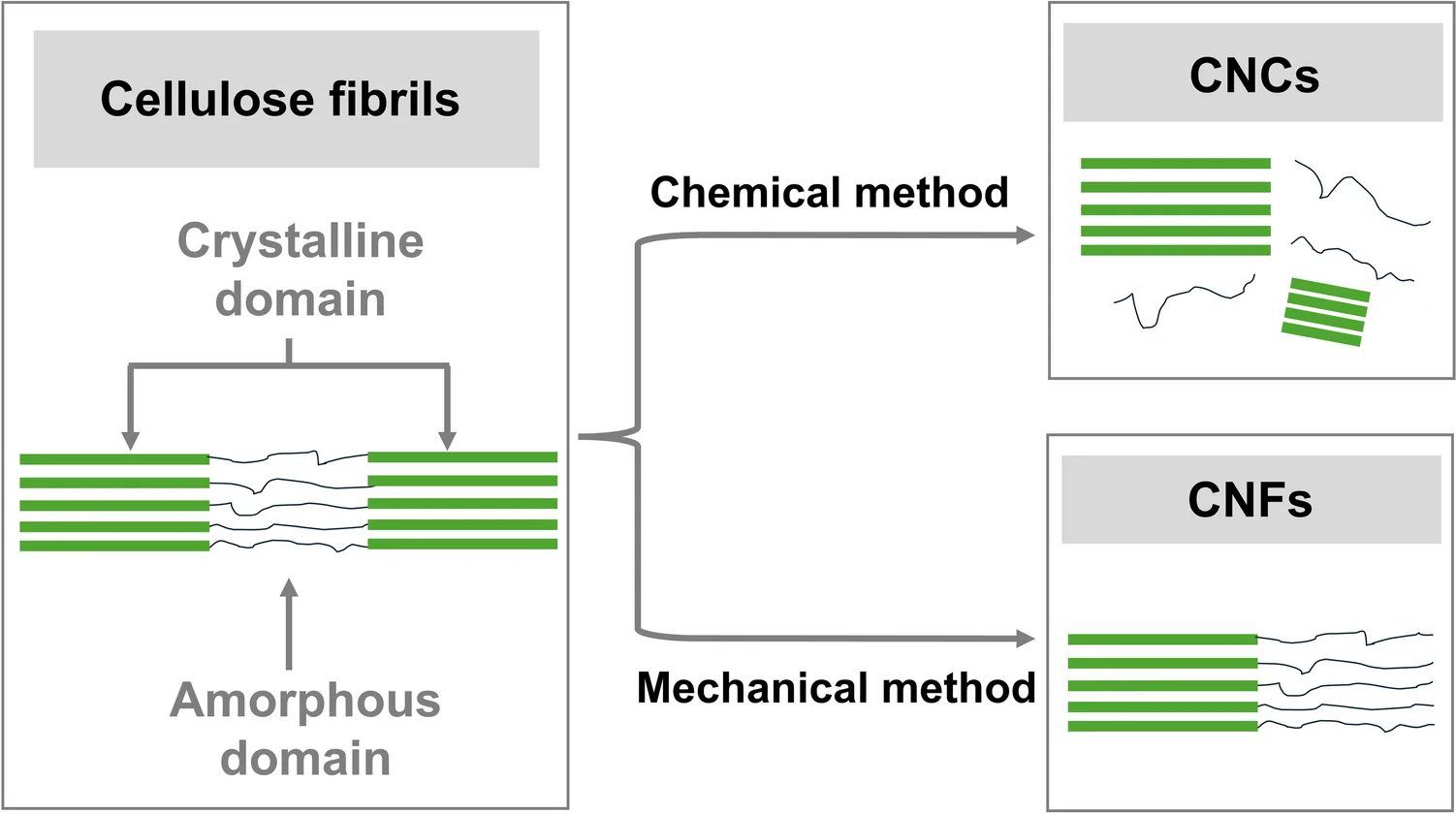
Schematic illustration of chemical and mechanical methods for producing CNCs and CNFs from cellulose fibrils

Cellulose, the most abundant renewable biopolymer, consists of alternating crystalline and amorphous regions that can be processed into nanoscale components. Chemical hydrolysis of crystalline regions produces rigid cellulose nanocrystals (CNCs), while mechanical processing separates amorphous regions into flexible cellulose nanofibrils (CNFs) (Fig. 8). Both retain cellulose's inherent biodegradability, hydrophilicity, and mechanical strength, but differ structurally and functionally. CNCs, with their rod-like morphology and high crystallinity, are ideal for passive antifouling surfaces such as hydration layers or water-repellent coatings. CNFs, characterized by their high aspect ratio and entangled network structure, are better suited for flexible, mechanically robust coatings with self-healing capabilities.

(i) CNCs

**Fig. 9:**
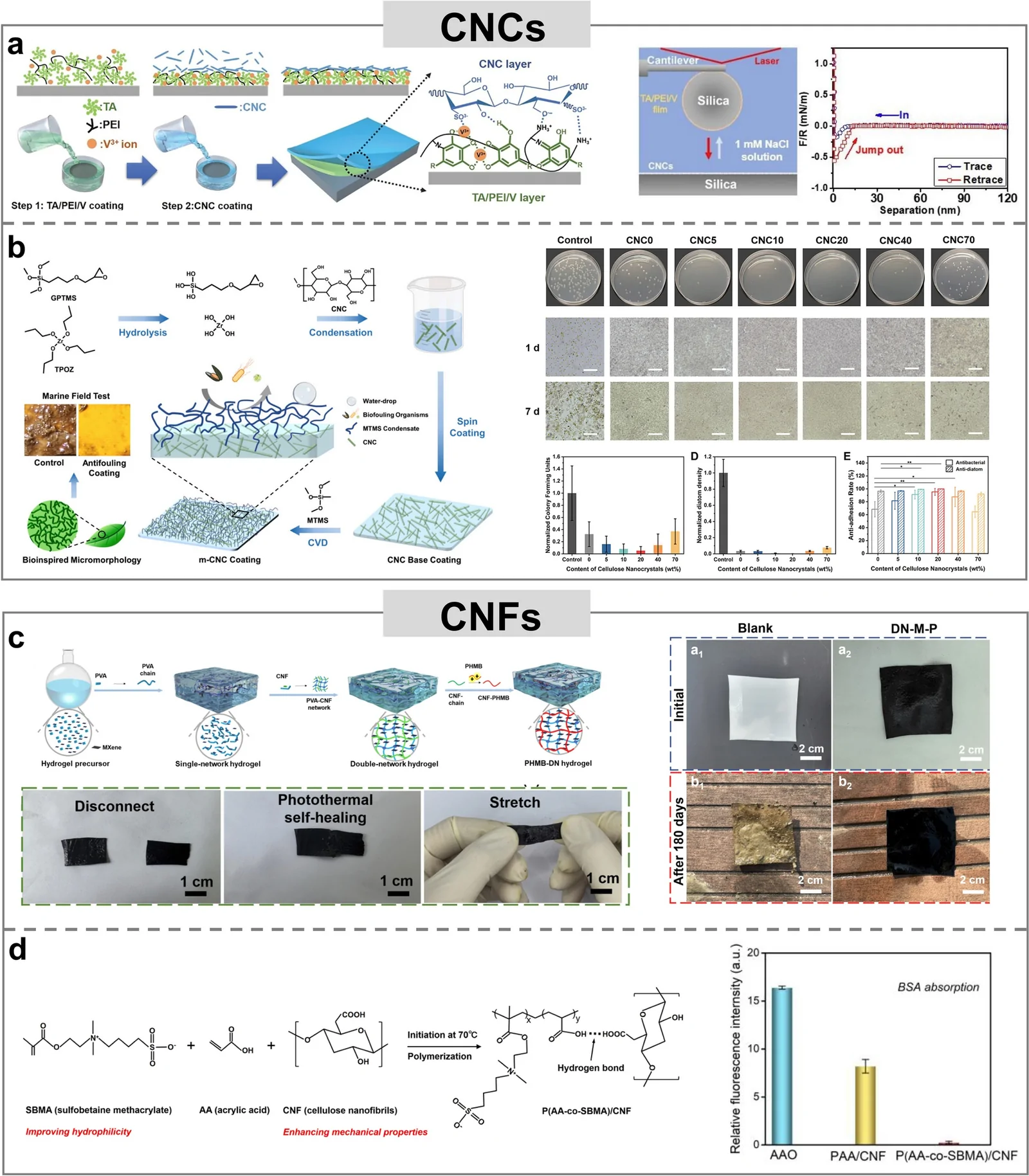
CNCs and CNFs based antifouling coatings. **a** Mussel-inspired TA/PEI/V interlayer for anchoring CNCs on substrates and AFM colloidal probe measurements showing the enhanced CNC–interlayer adhesion. Reproduced with permission from Ref. [210]. Copyright 2021, Wiley. **b** Bioinspired CNC-based coatings prepared via sol–gel condensation and their antibacterial and anti-diatom performance showing strongly reduced microbial attachment on CNC10 and CNC20 surfaces. Reproduced with permission from Ref. [211]. Copyright 2021, Elsevier. **c** CNF-based hydrogels with stretchable and photothermal self-healing properties and their long-term stability after 168 days in marine environments. Reproduced with permission from Ref. [212]. Copyright 2024, Elsevier. **d** Chemical structure of CNF-based coatings incorporating SBMA and AA and BSA adsorption tests demonstrating reduced protein fouling on the hybrid surfaces. Reproduced with permission from Ref. [213]. Copyright 2023, Elsevier

While CNCs can form stable hydration layers for antifouling, their weak substrate adhesion remains a major challenge. Inspired by mussel, Yang et al. developed a method of using the intermediate adhesive layer of tannin/polyethylene imide/vanadium (TA/PEI/V) to firmly anchor cellulose nanocrystals (CNCs) to various substrates [210], so as to obtain a dense and stable coating (Fig. 9a). Atomic force microscopy (AFM) colloidal probe measurement further reveals that there is a strong interface adhesion between the TA/PEI/V layer and CNCs (F/R ≈ 0.62 mN m^−1^). These superhydrophilic surfaces reduce the adsorption of bovine serum albumin (BSA) from ~ 700 to ~ 60 ng Hz^−1^ cm^−2^, and are easy to rinse, showing excellent anti-fouling performance. CNCs can also construct surface microtopography. Duan et al. mixed CNCs into the sol–gel siloxane matrix to replicate the ridge-like structure of mangrove leaves (Fig. 9b) [211]. In the antibacterial and diatom-adhesion tests, coatings containing CNCs, particularly CNC10 and CNC20, showed sharply reduced microbial attachment. This outcome demonstrates that CNC-regulated microstructures strengthen surface stability and lower organism–surface interactions, thereby enhancing early antifouling performance.

Beyond passive strategies, CNCs enable active biocide delivery. Zhou et al. developed CNC-reinforced silica coatings loaded with Econea, a biodegradable biocide, achieving controlled release over 28 days [214]. CNCs stabilized the silica matrix while regulating biocide diffusion, resulting in 99% bacterial inhibition and 90% diatom reduction. This demonstrates a promising strategy: integrating passive hydration barriers with active biocide release for sustained antifouling performance.

(ii) CNFs

CNFs offer complementary advantages to CNCs by enhancing mechanical strength and flexibility in antifouling coatings. Their entangled network structure reinforces coating integrity, improves adhesion, and enables self-healing capabilities. Xiong et al. mixed carbon nanofibers (CNF) into CNF-MXene hydrogel with polyvinyl alcohol (PVA) matrix (Fig. 9c) [212]. The high water-retention capacity of CNFs facilitated photothermal self-healing, allowing the coating to restore antifouling function under sunlight. This self-healing mechanism still maintains an antibacterial efficiency of 99.97% after six months of exposure to the ocean, showing how CNF extends the durability and functional life of the structure. Long et al. used different methods to graft CNF and SBMA to prepare a flexible, highly hydrophilic anti-fouling surface (Fig. 9d) [213]. The stable CNF bracket supports the amphoteric ion hydration layer, which can effectively resist the adhesion of proteins, bacteria, and diatoms, achieving a 98% biological pollution resistance rate.

These studies together show that CNFs are highly adaptable materials with both mechanical strength and functional flexibility. CNFs can be used as both structural support and reaction surface, so they can be used to prepare durable, self-healing, and environmentally friendly anti-fouling coatings. However, improving its water stability and making it easier for large-scale processing is still an important challenge in practical applications.

#### Alginate

Alginate is a natural polysaccharide that exists in the biofilm of algae and bacteria. Because of its strong water retention, excellent biocompatibility, and biodegradability, it is a promising marine anti-fouling coating material. Its hydrate layer helps to prevent the accumulation of protein and prevent the adhesion of marine organisms such as barnacle larvae and diatom spores. However, in seawater, due to the cross-linking action caused by divalent ions (such as Ca^2+^ and Mg^2+^), the anti-fouling ability of alginate will decrease, as divalent ions will reduce their hydration. Research on poly(AAm)/alginate hydrogels has also shown that, even when reinforced with multivalent ions, they still suffer from low mechanical strength and poor stability under ion-rich aqueous environments. This instability, caused by ion leaching, limits their long-term use [215].

**Fig. 10:**
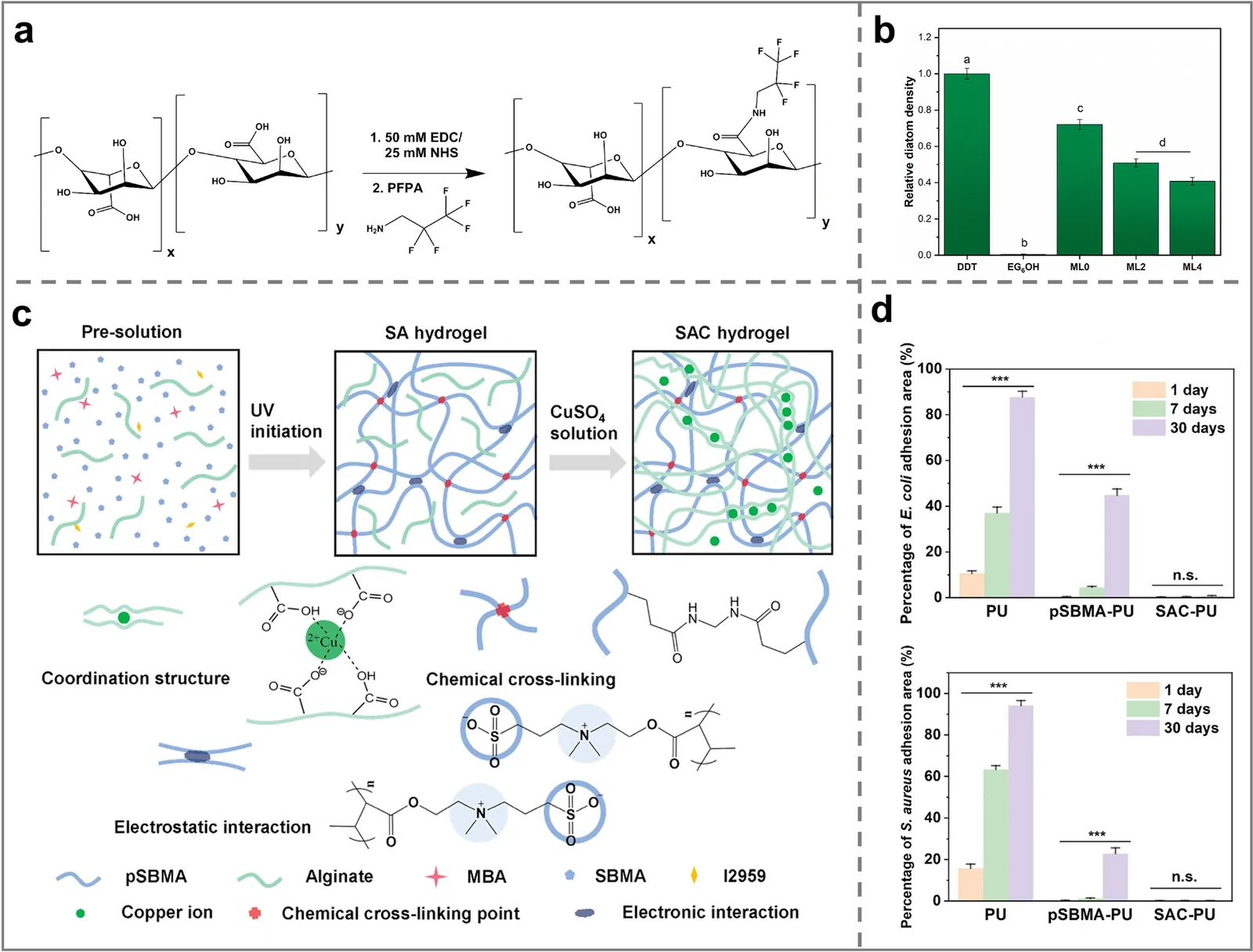
Alginate-based antifouling coatings. **a** Schematic illustration of alginate modification with PFPA to prepare amphiphilic coatings. **b** Evaluation of diatom and green algal spore adhesion on amphiphilic alginate coatings. Reproduced with permission from Ref. [65]. Copyright 2022, American Chemical Society. **c** Schematics of SAC hydrogel preparation processes. **d** Percentages of adhesion area of *E. coli* and *S. aureus* on the SAC coatings. Reproduced with permission from Ref. [216]. Copyright 2024, Wiley

In order to improve its stability and antifouling performance, researchers adopted chemically modified and composite coating strategies. Gnanasampanthan et al. prepared an amphiphilic coating by pentafluoro propylamine (PFPA) capping alginate (Fig. 10a) [65], which improved the antifouling ability while retaining the hydration properties, resulting in a reduction of diatom and green algal spore adherence by about 50% (Fig. 10b). However, the environmental concerns associated with fluorinated compounds have driven researchers to explore other alternative non-fluorinated modification strategies. Lee et al. addressed the instability of alginate coatings in seawater by anchoring an atom transfer radical polymerization (ATRP) initiator onto the carboxyl groups of alginate, followed by surface-initiated polymerization of SBMA, thereby forming a stable polymer brush structure [181]. The resulting multilayered alginate/poly(SBMA) coatings combined the intrinsic hydration capacity of alginate with the zwitterionic antifouling properties of poly(SBMA), leading to a synergistic effect. Even with a thin layer of ~ 20 nm poly(SBMA), diatom adhesion was reduced by 94% compared with alginate alone, demonstrating remarkable marine antifouling efficiency. In addition, Yan et al. developed a poly(SBMA)/alginate–Cu^2+^ (SAC) zwitterionic hydrogel coating on PU surfaces (Fig. 10c) [216], which achieved 81.5% inhibition of bacterial adhesion while enhancing the structural stability through the antimicrobial action of Cu^2+^ ions, thereby demonstrating excellent and durable antifouling performance (Fig. 10d). Although these optimization strategies enhanced the antifouling ability of alginate coatings, their long-term stability in complex marine environments remains a key challenge. Future research should further optimize the cross-linking method and develop environmentally friendly modification strategies.

### Protein

#### Gelatin

Gelatin, which is obtained through the partial hydrolysis of collagen, exhibits exceptional functional properties that render it valuable for antifouling applications, as summarized in Table 5. The inherent hydrophilicity of gelatin-based coatings has been shown to create surfaces with reduced protein adsorption and cell adhesion. Wang et al. exemplified this by developing a CS-AgNPs@PAAm-Gelatin nanocomposite coating on endotracheal tubes [217], where the PAAm-Gelatin network provided strong hydrophilicity and covalent bonding to the PVC substrate. This design effectively suppressed protein, cell, and platelet adhesion while the embedded silver nanoparticles contributed antibacterial functionality, demonstrating the dual antifouling and antibacterial capacity of gelatin-based systems. Unlike this strategy, which was based on hydration, Li et al. prepared gelatin oxide pectin aerogel modified with PDMS and silver nanowires [218], indicating that the hydrophobic barrier can also reduce the adhesion of pollutants while providing antibacterial components.

When combined with inorganic nanoparticles, gelatin shows enhanced dirt resistance. Lu et al. mixed CoFe_2_O_4_ magnetic nanoparticles into the gelatin matrix to construct a structured surface with strong resistance to biofouling [219]. Gelatin acts as an effective carrier of capsaicin through hydrogen bonding, achieving a high dose of 92 mg g^−1^, and maintaining coating stability after 21 days, with very little hydrophobic change. These findings highlight the dual role of gelatin as a biocompatible scaffold and an active carrier of antibacterial agents. Future design may further use the controllable network chemical properties of gelatin to realize a controlled release or a multi-responsive antifouling mechanism other than passive adsorption.

#### Keratin

Keratin is a fibrous structural protein derived from wool and hair, which has unique advantages in antifouling coating. Keratin possesses zwitterionic characteristics and exhibits strong hydration capacity, rendering it highly compatible with polysulfobetaine-based systems. Shang et al. integrated keratin into a polysulfobetaine dual-network hydrogel, demonstrating enhanced hydration properties, reduced BSA adsorption, and superior antibacterial activity. These findings underscore keratin’s promise as a hydration-enhancing agent for antifouling coating applications [223]. Similarly, Atrian et al. reported that electrospraying keratin coating on the silk protein-Laponite membrane can enhance its hydrophilicity and antibacterial properties while maintaining mechanical integrity [226].

Overall, these studies demonstrate that keratin is a biofunctional antifouling material capable of providing both passive resistance and active biological interaction. However, to fully realize its potential, it is essential to precisely regulate its molecular structure, degradation rate, and interface activity in order achieve an optimal balance between antifouling durability and biological responsiveness.

#### Silk Fibroin

Silk fibroin (SF), extracted from silkworm cocoons, possesses a unique hierarchical structure composed of crystalline *β*-sheet domains interspersed with amorphous regions, giving it excellent processability for fabrication into thin films, fibers, and porous scaffolds. These structural characteristics allow SF to be engineered with micro-nanostructures that effectively resist bacterial adhesion and biofilm formation. For example, Xie et al. developed an SF-based catheter with a lotus leaf-like micromastoid surface that exhibited strong resistance to *E. coli* and *S. aureus* colonization [225]. Similarly, Li et al. reported a superhydrophobic SF/Ag nanowire composite membrane capable of effectively repelling low-surface-tension liquids [227].

Overall, these studies highlight SF as a robust platform for designing durable antifouling surfaces. Its hierarchical organization and tunable secondary structure confer both stability and design versatility. Future work could further exploit these properties to develop adaptive antifouling interfaces responsive to environmental stimuli.

**Table 5:** Summary of protein-based antifouling coatings

Biomass Type	Formulation	Antifouling mechanism	Antifouling performance	References
Gelatin	CS/MPA-BN^+^SO_3_^−^/gelatin	Contact-killing	Minimum Inhibitory Concentration (MIC):	[220]
			*E. coli:* 22 mg mL^−1^,	
			*S. aureus:* 5.5 mg mL^−1^	
Gelatin	MXene/Liposomes/gelatin	Hydrophilic layer, contact-killing	Adhesion rates:	[221]
			*S. aureus:* 2.5%	
Gelatin	OXG/GS/gelatin	Release-killing	Antibacterial rate:	[222]
			*E. coli:* 99.99%,	
			*S. aureus:* 99.99%	
Keratin	Chlorhexidine/pSB/keratin	Hydrophilic layer, release-killing	FITC-labeled BSA adsorption:	[223]
			no protein was found	
Silk fibroin	Silk fibroin	Wrinkled surfaces	Biofilm coverage area:	[224]
			*E. coli:* 0.3% (wrinkled), 42.7% (unwrinkled), 82.3% (uncoated),	
			*S. aureus:* 3.5% (wrinkled), 67.3% (unwrinkled), 90.3% (uncoated)	
Silk fibroin	Silk fibroin	Mimicking lotus leaf microstructures	Crystal violet staining (Absorbance):	[225]
			*E. coli:* ~ 0.1,	
			*S. aureus:* ~ 0.2	

Chitosan (CS), Maleopimaric acid (MPA), Oxidized xanthan gum (OXG), Gentamicin sulfate (GS), Polysulfobetaine (pSB)

### Comparative Evaluation of Biomass-Based Coatings

Despite their diverse structures, the biomass categories discussed share fundamental characteristics: (1) high functional group density facilitating chemical modification; (2) intrinsic biodegradability and low environmental toxicity; and (3) a renewable nature with a low carbon footprint. However, a common observation emerges across these materials: antifouling performance is not governed by a single function but rather by the delicate balance among hydration capacity, structural integrity, and interfacial stability. As summarized in Table 6, each biomass category presents distinct advantages and inherent limitations. Zwitterionic systems exhibit excellent resistance to protein and cell adhesion, however, their soft and highly hydrated networks often lack the robustness required to endure long-term mechanical stress. Lignin and tannins impart chemical stability and rigidity, yet their inherent hydrophobicity or heterogeneity can compromise the uniformity of the antifouling interface. Polysaccharides, such as chitosan and cellulose derivatives, offer favorable hydration and adhesion, but their tendency to swell and sensitivity to environmental fluctuations restrict long-term durability. Similarly, protein-based coatings demonstrate high biocompatibility but remain prone to rapid degradation unless stabilized through advanced crosslinking.

**Table 6:** Summary of the strengths, limitations, and representative application scenarios of biomass-based materials

Materials	Strengths	Limitations	Applications
Lignin	High stiffness, UV stability, antioxidant activity, low cost	Brittle; broad molecular weight distribution; strong solvent dependence	Mechanical reinforcement, UV-resistant coatings, hybrid foul-release networks
Tannin	Strong metal coordination, inherent antimicrobial activity	Prone to leaching; metal-ion exchange causing network collapse	Antimicrobial membranes, medical devices, coatings requiring moderate hydrophilicity
Betaine	Exceptional hydration layer, superior resistance to proteins and biofilms	Weak mechanical strength, poor wear resistance, low adhesion	Biomedical interfaces, catheter coatings, antifouling layers for optical sensors
Chitosan	Intrinsic antibacterial activity, strong adhesion, reactive amine groups	pH-sensitive solubility; brittle networks; accumulation of dead cells facilitating new biofilm formation	Antimicrobial medical coatings, adhesive biomaterial coatings, hybrid antibacterial networks
CNCs/CNFs	High mechanical reinforcement, hydrophilicity, bioinspired structuring	CNCs: weak substrate adhesion; CNFs: long-term water instability	Structural marine coatings, flexible antifouling films, bioinspired textured surfaces
Proteins	Biocompatible, tunable structure, easy processing	Prone to biodegradation; thermally and moisture sensitive	Biodegradable medical devices, protein-resistant coatings, controlled-release surfaces

These comparisons highlight that the principal challenge lies not in a shortage of effective antifouling mechanisms, but in the seamless integration of these mechanisms into a unified material system. Therefore, future advances will rely on the design of hybrid architectures that reconcile hydration capacity with mechanical toughness, thereby achieving sustained performance under practical service conditions.

## Applications of Biomass-based Antifouling Coatings

Application-specific requirements directly guide the selection and modification of biomass materials. In marine environments, where coatings must withstand high salinity and mechanical abrasion, robust materials like lignin and tannins are preferred. These polyphenolic biomolecules exhibit strong underwater adhesion, antioxidant capacity, and natural antimicrobial activity. Their performance can be further enhanced through covalent crosslinking or metal-coordination, which improves mechanical durability and ionic resistance while maintaining redox stability and fouling resistance [228–230]. In contrast, biomedical applications prioritize biocompatibility and hemocompatibility [231], leading to the selection of highly hydrophilic or zwitterionic materials such as polysaccharides, betaines, and proteins. These materials are typically modified via surface grafting or light crosslinking to preserve hydration-layer dynamics and chain mobility while ensuring non-toxicity. Such application-driven design allows biomass-based coatings to reconcile environmental constraints with specific antifouling targets, ranging from ship hulls to delicate medical implants, demonstrating the adaptability and functional diversity of biomass materials.

### Marine Applications

#### Ship Hulls

**Fig. 11:**
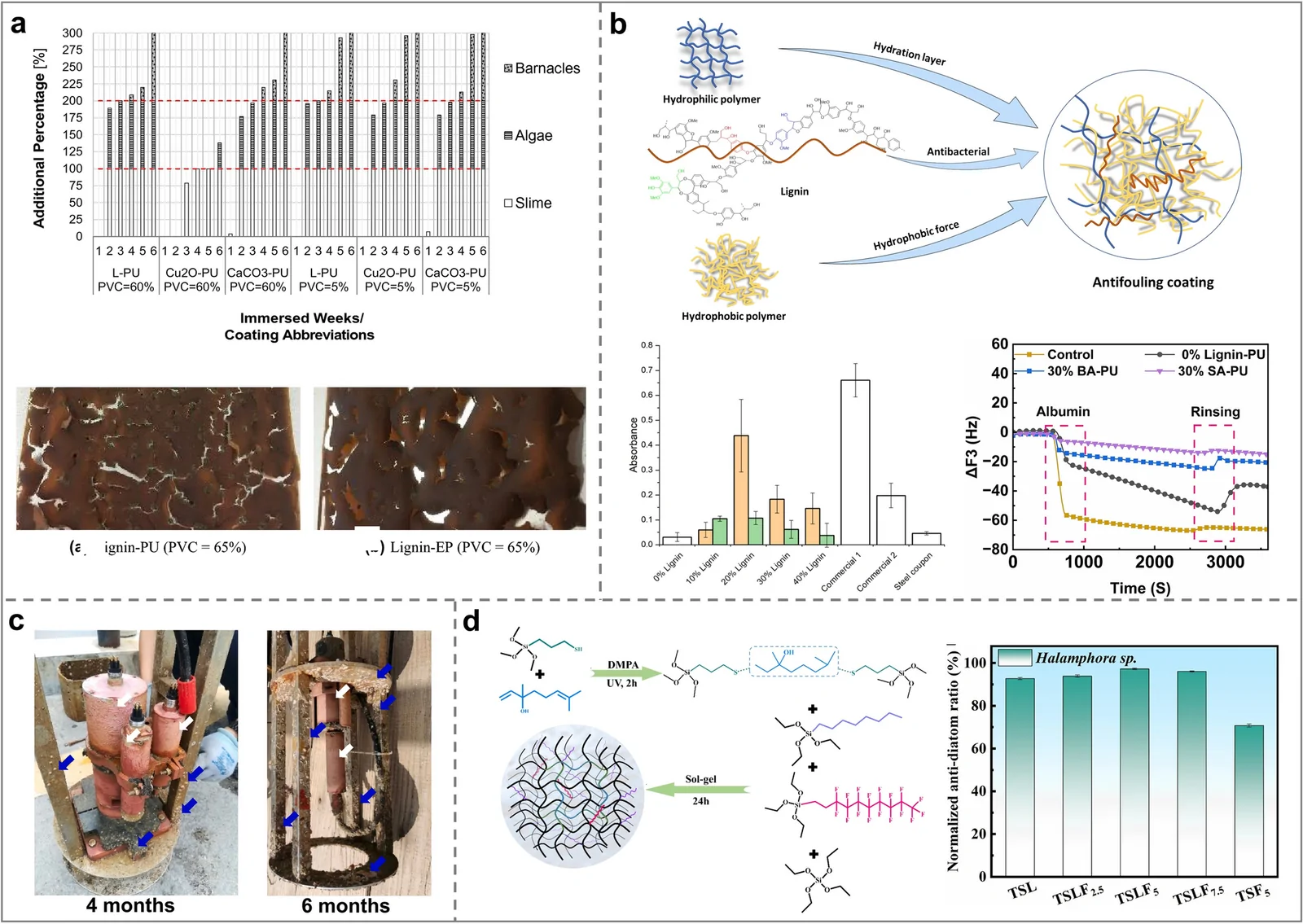
Biomass-based antifouling coatings used in marine applications. **a** Antifouling performance of lignin-PU coatings during field immersion and the poor cohesion of brittle lignin films. Reproduced from Ref [232]. under the provisions of the CC BY 4.0 License. **b** Design strategy of organosolv lignin-PU antifouling coatings and corresponding bacterial-adhesion and QCM-D analysis of BSA adsorption results for different lignin-containing formulations. Reproduced from Ref [162]. under the provisions of the CC BY 4.0 License. **c** Application of CPT-based coating on the housings of the COD sensor and the Chl* a* sensors, as well as their wipers, for 4 months, and on the housing of the BOD sensor for 6 months (White arrows indicate CPT-coated surface; blue arrows indicate uncoated frames). Reproduced with permission from Ref. [233]. Copyright 2022, Elsevier. **d** Synthesis of linalool-based transparent coatings and their normalized anti-diatom performance. Reproduced with permission from Ref. [234]. Copyright 2024, Elsevier

In the complex marine environment encountered during ship navigation, hull coatings must not only exhibit excellent antifouling properties, but also provide superior wear resistance and strong adhesion to withstand seawater erosion, wave impact, and collisions with floating debris. To address these challenges, the limitations of using a single biomass-derived polymer in terms of mechanical performance and stability have led researchers to focus on hybrid strategies that integrate bio-based functional components with high-strength coating matrices. For example, Butschle et al. incorporated unmodified kraft lignin powder into waterborne polyurethane and epoxy systems to develop a bio-based antifouling coating [232]. Although the phenolic hydroxyl groups in lignin exhibit some antimicrobial activity in laboratory tests, long-term field exposure on ship panels showed limited antifouling performance (Fig. 11a). In particular, increasing the lignin content to enhance biological contact significantly deteriorated the mechanical properties of the coating, leading to brittleness and reduced adhesion. This result clearly indicates that simply adding more filler without ensuring adequate compatibility may compromise the structural integrity of the coating.

In contrast, Wu et al. proposed a more engineering-oriented approach [162]. They extracted highly acetone-soluble organosolv lignin from forestry residues and prepared a green antifouling coating by blending it with polyurethane at a controlled hydrophilic-PU to hydrophobic-PU ratio of 2:8 (Fig. 11b). Rather than simply mixing the bio-fillers into the matrix, their design ensured molecular-level compatibility by integrating lignin as a functional filler evenly dispersed within the polyurethane network, thereby avoiding the loss of mechanical strength. The coating exhibited enhanced water repellency, a higher nanoindentation modulus, and reduced friction. It also showed stronger scratch resistance and superior mechanical performance, achieving antifouling results that surpassed commercial Cu_2_O-based coatings. QCM-D measurements and biofilm assays with marine bacteria further confirmed its effective resistance to fouling.

Overall, the advancement of biomass-based marine coatings requires more than improving surface antifouling properties. It also demands optimization of the internal architecture to ensure mechanical integrity under marine conditions. A key challenge lies in balancing soft, hydrophilic antifouling domains with rigid, load-bearing components while maintaining strong interfacial adhesion. Future developments should prioritize hierarchically engineered composite designs that integrate elasticity, toughness, and antifouling functionality to deliver durable, long-term performance in dynamic marine environments.

#### Marine Sensors and Optical Windows

Marine sensors such as pressure, temperature, salinity monitors, and optical cameras often operate for extended periods without maintenance. Biofouling accumulates over time, reducing data quality and precision. Unlike ship hulls, these sensors contain delicate components like dissolved oxygen membranes, pH electrodes, and optical lenses that cannot tolerate harsh cleaning or toxic antifouling coatings [235–237].

The key challenge is preventing microbial adhesion without compromising sensor function [238]. Effective coatings must be ultrathin, chemically stable, seawater-compatible, and resistant to corrosion and not interfere with the measurement. Various antifouling strategies exist, including coatings, mechanical scrapers, UV light, and electrochemical pulses [54]. However, most of them require continuous power or maintenance. Only passive coatings can provide long-term fouling resistance without external intervention, making them ideal for remote deployments. The effectiveness of scrapers is limited by biofouling on the scrapers themselves, the mechanical reliability of components, and the physical complexity of sensor surfaces [239]. Electrochemical antifouling typically requires continuous or periodic application of current or voltage [240]. UV irradiation methods need optimization of parameters such as wavelength, exposure time, and duty cycle based on environmental conditions [241, 242]. Therefore, considering all factors, coatings represent an excellent strategy for combating biofouling and protecting marine sensors [243]. To address sensor-surface biofouling, Hao et al. evaluated a camptothecin (CPT)-based antifouling paint on six common sensor-housing materials (316L stainless steel, TC4 titanium alloy, 7075 aluminum alloy, polyoxymethylene, polyvinyl chloride, and PTFE), showing markedly lower macrofouling on painted versus unpainted areas over 9 months of seawater immersion. In a buoy-based sea trial, the CPT paint kept the housings of chemical oxygen demand (COD) and chlorophyll *a* (Chl *a*) sensors clean for more than 4 months, and the biochemical oxygen demand (BOD) sensor housing was free after 6 months (Fig. 11c), demonstrating strong potential for long-term marine monitoring applications [233].

Considering that optical window sensors have higher transparency requirements, Zhang et al. used linalool [234], which has natural antimicrobial properties, to construct a highly transparent biomimetic coating that achieved over 95% inhibition against typical fouling organisms without affecting visible light transmittance (Fig. 11d), making it suitable for underwater equipment requiring visual observation, such as cameras and fluorescence probes. Additionally, Li et al. reported an ultra-thin hydrogel-polymer composite coating with a thickness of only tens of nanometers [244], possessing excellent structural stability and resistance to water swelling, showing promise for application to underwater sensor components that are highly sensitive to coating thickness and surface response, providing new insights for material integration.

### Medical Applications

#### Medical Devices and Implants

**Fig. 12:**
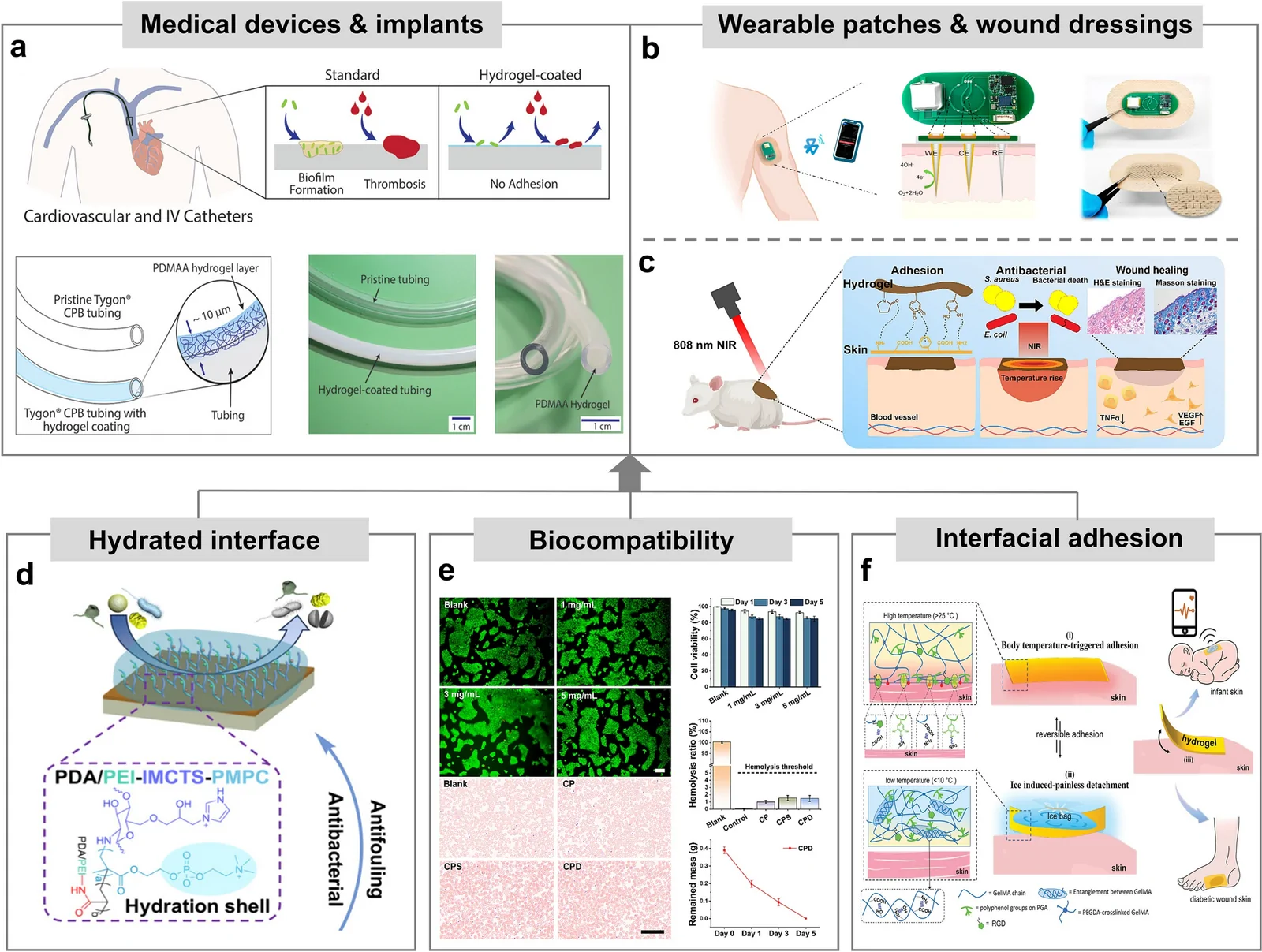
Biomass-based antifouling coatings used in medical applications. **a** Hydrogel coatings for medical devices, showing reduced biofilm formation and fouling on coated tubing compared with pristine surfaces. Reproduced with permission from Ref. [246]. Copyright 2020, Wiley. **b** Wearable hydrogel patches and wound dressings. Reproduced with permission from Ref. [247]. Copyright 2025, American Chemical Society. **c** NIR-responsive hydrogels for antibacterial and wound-healing use. Reproduced with permission from Ref. [248]. Copyright 2024, Elsevier. **d** Hydrated interface formed by hydrophilic coating. Reproduced with permission from Ref. [249]. Copyright 2024, American Chemical Society. **e** Biocompatibility evaluation by live/dead staining and hemolysis assay. Reproduced with permission from Ref. [248]. Copyright 2024, Elsevier. **f** Temperature- and ice-triggered interfacial adhesion of hydrogel patch. Reproduced with permission from Ref. [250]. Copyright 2022, American Chemical Society

Biofouling on implantable medical devices leads to infection, thrombosis, and functional failure (Fig. 12a). Effective coatings should create hydrated and charge-neutral interfaces that suppress nonspecific adsorption of proteins, cells, and microorganisms. Hydrophilic polymer networks based on polyethylene glycol (PEG) or zwitterionic polymers are widely applied to achieve this goal. These materials form dense hydration layers that prevent interfacial denaturation. Zwitterionic polymers, such as carboxybetaine (CBMA) and sulfobetaine (SBMA), show lower hydration free energy and stronger resistance to fouling compared with PEG [245].

In practice, biomass-derived substrates like polyurethane modified with bioinspired adhesive primers (e.g., polydopamine, PEI) have shown notable potential for medical device coatings. Wei et al. fabricated a PDA/PEI-based ternary copolymer containing sulfobetaine and quaternary ammonium units [91], achieving both antifouling and bactericidal effects. Similarly, Huang et al. grafted chitosan derivatives and PMPC copolymers onto PU surfaces (Fig. 12d) [249], creating dual-function coatings with strong bacterial inhibition and hemocompatibility. To meet electronic implant requirements, Wellens et al. constructed an ultra-thin PDA/PEI/Poly(SBMA-co-MA) film (~ 6 nm) [251], which effectively suppressed protein and cell adhesion while maintaining stable electrical impedance. Collectively, these biomass-based and zwitterionic hybrid coatings highlight a general antifouling principle for medical implants: maintaining a stable hydration layer and charge-neutral surface to resist bio adhesion without compromising interfacial function or electrochemical performance.

#### Wearable Patches and Wound Dressings

Beyond implantable systems, antifouling coatings are increasingly required for wearable patches and wound dressings (Fig. 12b, c), where moist, protein-rich interfaces pose challenges to safety and comfort. The design focuses on integrating biocompatibility, reversible adhesion, and environmental stability within biomass-based networks. Recent studies have explored mussel-inspired polysaccharide networks capable of maintaining hydration while exhibiting antimicrobial and photothermal properties. For instance, Ouyang et al. developed a dopamine-modified alginate/chitosan/PVP hydrogel coating [248], whose catechol-rich network provided strong interfacial adhesion and near-infrared activated antibacterial capability. The coating demonstrated excellent cell viability and hemocompatibility (Fig. 12e), confirming its biocompatibility. This bio-derived catechol chemistry approach effectively combines antifouling performance with biological safety. Similarly, Manoharan et al. incorporated green-synthesized cerium oxide nanoparticles into chitosan-alginate films, creating nanostructured composites with combined antioxidant and antimicrobial properties [252].

Beyond passive hydration, dynamic self-regulating hydrogels have emerged as advanced antifouling systems. Cheng’s group developed an oxidized alginate-gelatin network with reversible Schiff-base bonds, enabling self-repair and moisture retention through glucose oxidase-catalase feedback [253]. This system autonomously adjusts pH and bonding strength to maintain stable hydration under varying conditions. Jiang et al. explored temperature-responsive control by designing a protein–polyphenol network that adheres at body temperature but releases upon cooling (Fig. 12f).

These advances represent a shift from static barriers to adaptive interfaces. However, the enzymatic feedback loops and thermal-responsive mechanisms require relatively stable conditions that may not exist in complex marine environments. The long-term reliability of such dynamic systems under continuous biofouling pressure remains uncertain, suggesting that simpler passive coatings may still offer more practical solutions for remote sensor deployments.

### Emerging and High-Performance Applications

Beyond the traditional marine and biomedical sectors, the design principles of biomass-based antifouling coatings are increasingly relevant to high-performance and safety–critical industries. In aviation and aerospace fuel systems [254], biomass-derived interfaces can mitigate the accumulation of microbial biofilms and metabolic by-products that cause fuel degradation and sensor failure. Similarly, the integration of ultra-thin, hydration dominated layers is crucial for electrochemical sensors and precision analytical instruments [255–257]. In these systems, even trace amounts of nonspecific bio adhesion can disrupt fluid flow, increase electrical impedance, or mask active electrode sites, thereby significantly compromising signal transduction and sensing sensitivity.

In energy-related infrastructures, such as solar desalination membranes and heat exchangers [258–260], biomass-based coatings providing hydrophilic surfaces or incorporating active antimicrobial elements like silver nanoparticles offer a sustainable solution to combat fouling while maintaining high thermal and operational efficiency [161]. These emerging examples highlight the broad transferability of biomass materials, as their tunable surface chemistry and mechanical integrity allow for tailored protection in complex and demanding environments.

## Challenges

### Mechanical Durability

**Fig. 13:**
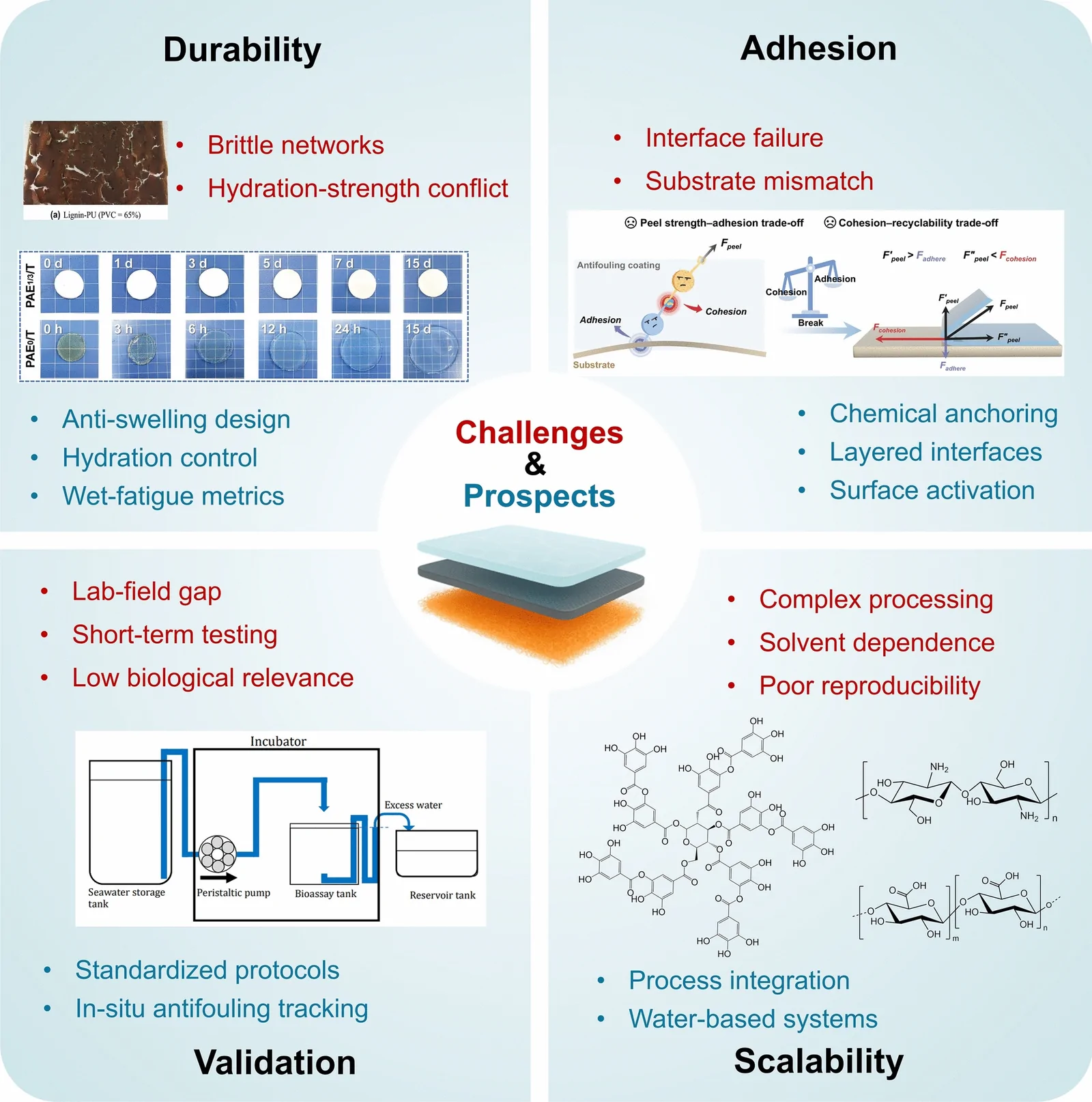
Challenges and prospects of biomass-based antifouling coatings [232, 273–275]

Biomass-derived coatings usually have poor mechanical durability due to the softness or brittleness of the polymer network (Fig. 13) and are prone to cracking, deformation, and wear under the action of shear stress or cyclic load [261]. Water-induced swelling generates internal stress that accelerates degradation [262, 263], while environmental factors such as UV radiation and temperature fluctuations cause polymer chain scission and fatigue [264]. Strategies such as optimized network architectures [265, 266], nanofiller reinforcement [267, 268], and dynamic cross-linking can improve the toughness and self-healing ability of the coating [212, 269]. However, it is still a challenge to balance the mechanical durability and anti-fouling performance of the coating under continuous water-based conditions [270–272].

The key bottleneck lies in the inherent trade-off between the antifouling performance, which depends on hydration and mechanical integrity. Hydrophilic coatings that resist biological contamination through water binding are usually susceptible to reduced strength caused by swelling. Future designs may need to decouple these properties through a layered structure or adaptive system, so as to regulate hydration under mechanical stress. It is necessary to formulate standardized test procedures for wet wear, fatigue life, and stiffness retention to establish performance benchmarks and guide coating development.

### Adhesion and Interfacial Compatibility

Even antifouling coatings with high mechanical strength may fail due to poor substrate adhesion [276, 277]. The penetration, swelling, and ion exchange of water will gradually weaken the interface bond, leading to foaming and stratification. The diversity of substrates exacerbates this challenge, because metals, ceramics, polymers, and elastomers each have different interfacial chemical properties [210, 278, 279]. Researchers have adopted a variety of strategies to enhance adhesion, including silane coupling agent [280–282], catechol-functionalized primers [115, 283, 284], and interface mechanical interlocking through covalent bonds, coordination bonds, or hydrogen bonds [285]. However, these methods tend to fail under the action of long-term water exposure or cyclic mechanical load.

The adhesion problem highlights a core defect in coating design: interface engineering is often regarded as a secondary factor, although it is a common point of failure. Most studies focus on the overall performance of the coating and ignore the substrate-coating interface that usually fails. Future research and development should adopt a more comprehensive approach to combine interface chemistry and mechanical design. For example, the multi-layer structure that combines the rigid anchoring layer with the flexible top surface can improve the long-term stability of the coating.

### Field and Clinical Validation

One of the key challenges that research and development of anti-fouling coatings facing is the disconnect between laboratory performance and practical application performance. Most studies use static or short-term tests to capture the synergistic effects of factors such as shear stress, biofilm maturity, and environmental changes [286]. Therefore, coatings with excellent laboratory performance have poor durability or poor stability in field tests [287, 288]. For marine applications, verification must go beyond simple immersion tests, including liquid pool tests, near-shore test plate tests, and long-term deployment on buoys or ships [54, 289–291]. For biomedical applications, it is very important to combine in vitro testing with in vivo validation [292, 293], because the formation of protein coronal structure [294, 295], immune activation, and other host-material interactions will significantly change the surface anti-fouling performance or biocompatibility of the coating [296].

This validation gap reflects a deeper methodological problem: the absence of standardized protocols that predict long-term performance. Current laboratory tests prioritize convenience over biological relevance, yielding performance assessments that do not translate to real conditions. The field needs test conditions reproducing critical failure modes, such as wet-dry cycles, biofilm succession, and mechanical abrasion. Without such standards, coating development remains empirical, and innovations fail unpredictably in real environments.

### Scalability and Commercialization Barriers

Scaling biomass-based coatings to industrial production faces significant challenges. Natural materials such as lignin [297–299], tannin [300], and chitosan exhibit substantial variability in molecular weight, purity, and functional groups, resulting in inconsistent coating performance [301–303]. This heterogeneity, while enabling functional diversity, fundamentally undermines manufacturing reproducibility. Industrial processing demands narrow molecular weight distributions and predictable reactivity, suggesting that future biomass coatings may require fractionation or modification steps that sacrifice green credentials for processability. Additionally, many formulations rely on organic solvents, complex curing protocols, or unstable intermediates incompatible with sustainable manufacturing.

To bridge the gap between laboratory research and industrial application, practical strategies for the large-scale production of biomass coatings must be prioritized. First, standardized raw material processing is essential to mitigate the inherent batch-to-batch variability of natural biomass (e.g., lignin or tannins) [304–308], ensuring consistent chemical functionality and coating performance. Second, developing low-cost and “green” synthesis routes, such as water-based formulations or solvent-free processes, can significantly reduce both economic costs and environmental footprints. Furthermore, the transition from batch synthesis to continuous manufacturing approaches, such as roll-to-roll coating or large-scale spray-assisted assembly, represents an important step toward achieving uniformity over large surface areas. Collectively, these process-oriented strategies are expected to improve the reliability and scalability of biomass-based antifouling technologies for practical deployment.

### Environmental Impact and Sustainability Considerations

Although biomass-based antifouling coatings are often considered environmentally benign due to their renewable origins, their long-term environmental impact after application requires careful evaluation. In practice, degradation behavior and environmental persistence are strongly influenced by chemical modification, crosslinking density, and composite architecture, rather than by biomass feedstock alone. Current studies primarily emphasize short-term antifouling efficacy, while the long-term degradation kinetics, metabolic by-products, and ecological safety remain less explored. For instance, highly crosslinked or hybrid biomass systems, while mechanically robust, may exhibit significantly reduced biodegradability, highlighting a potential trade-off between operational durability and post-service sustainability. This “natural-but-persistent” paradox necessitates a move beyond the simple assumption that biological origin equates to absolute ecological safety.

From a sustainability perspective, future research should integrate life-cycle assessment (LCA) frameworks that evaluate antifouling coatings from “cradle to grave”, considering efficacy alongside mineralization pathways and end-of-life behavior. Establishing standardized protocols for assessing biodegradation, leaching, and bioaccumulation under realistic environmental conditions will be critical for regulatory translation and meaningful performance comparison. Looking ahead, promising directions include design-for-degradation strategies, such as the use of stimuli-responsive or dynamic covalent linkages that decouple service-life stability from long-term persistence. Such "smart" architectures ensure the coating remains stable during its functional period but triggers rapid degradation upon exposure to specific environmental triggers, truly aligning biomass-based technologies with global sustainable development goals.

## Summary and Outlook

This review provides a comprehensive overview of recent advances in biomass-based antifouling coatings, with emphasis on antifouling mechanisms, synthesis strategies, material selection, and application scenarios. By systematically examining representative biomass derived materials including polysaccharides, betaines, lignin, tannins, and proteins, we highlight how intrinsic chemical features such as hydrophilicity, charge regulation, and dynamic bonding capability govern interfacial antifouling behavior. Particular attention is given to the integration of passive and active antifouling mechanisms, the role of crosslinking and network architecture, and the emerging challenges associated with mechanical durability, scalability, and environmental sustainability. Together, this review underscores the growing potential of biomass-based coatings as environmentally compatible alternatives to conventional antifouling technologies, while also revealing critical knowledge gaps that must be addressed for practical deployment.

Despite significant progress, several key scientific and translational challenges remain. Future research should further strengthen mechanistic structure function linkages to enable rational antifouling design beyond empirical formulation driven approaches, particularly under realistic service conditions involving abrasion, salinity fluctuations, and mixed species fouling. Hierarchical and composite coating architecture are expected to play an increasingly important role in mitigating the long-standing trade-off between mechanical robustness and antifouling efficiency, while preserving manufacturability and cost effectiveness. In parallel, sustainability considerations must extend beyond renewable feedstocks to include degradation pathways, leaching behavior, and post application ecological safety, motivating life cycle-oriented evaluation and design for degradation strategies.

Looking forward, emerging opportunities lie in multifunctional and responsive biomass-based coatings, where interfacial properties can be dynamically regulated by environmental stimuli such as pH, ionic strength, or mechanical stress to adapt to evolving fouling conditions. Beyond responsiveness, increasing attention is expected to focus on hierarchical architecture, interface specific design, and decoupling strategies that independently optimize antifouling efficiency, mechanical durability, and environmental persistence. Notably, the integration of antifouling functionality with online fouling monitoring and self-reporting capabilities represents a promising frontier. In parallel, data driven and computational approaches, including machine learning-assisted material optimization, are anticipated to accelerate discovery by establishing predictive structure processing performance relationships across diverse biomass systems. Collectively, these directions point toward a new generation of biomass-based antifouling coatings that are not only effective and durable, but also adaptive, intelligent, scalable, and truly sustainable.
